# Supplementary material for: Experiences of individuals with rheumatoid arthritis interacting with health care and the use of a digital self-care application: a qualitative interview study
Source: BMJ Open. 2023 Dec 20;13(12):e072274. doi: 10.1136/bmjopen-2023-072274 (PMC10748980; doi:10.1136/bmjopen-2023-072274)
Supplement: Supplementary data [file bmjopen-2023-072274supp001.pdf]

Example of App Content: Explore snapshots of the app's features. For more details, please visit: https://www.elsa.science/en/

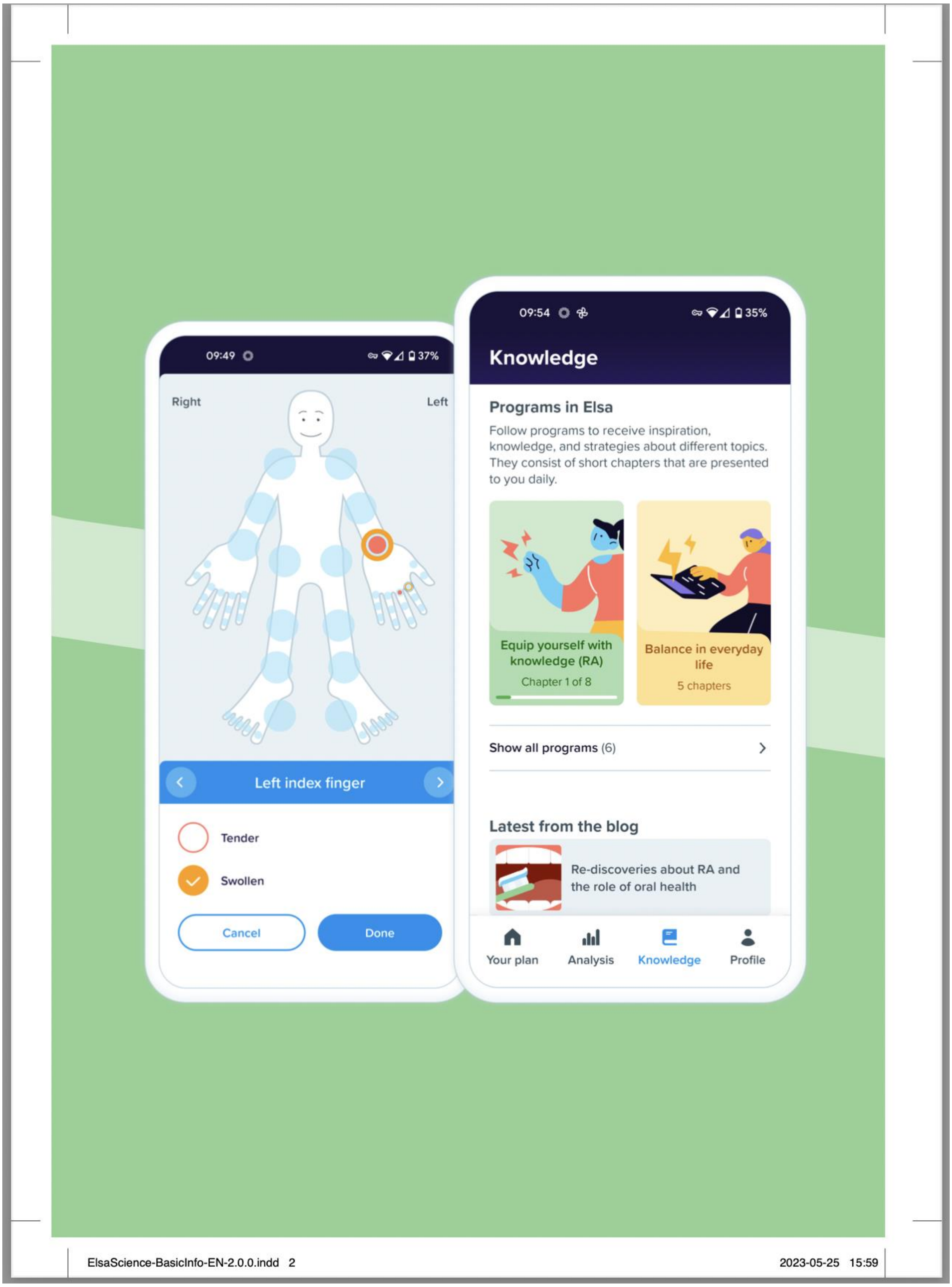

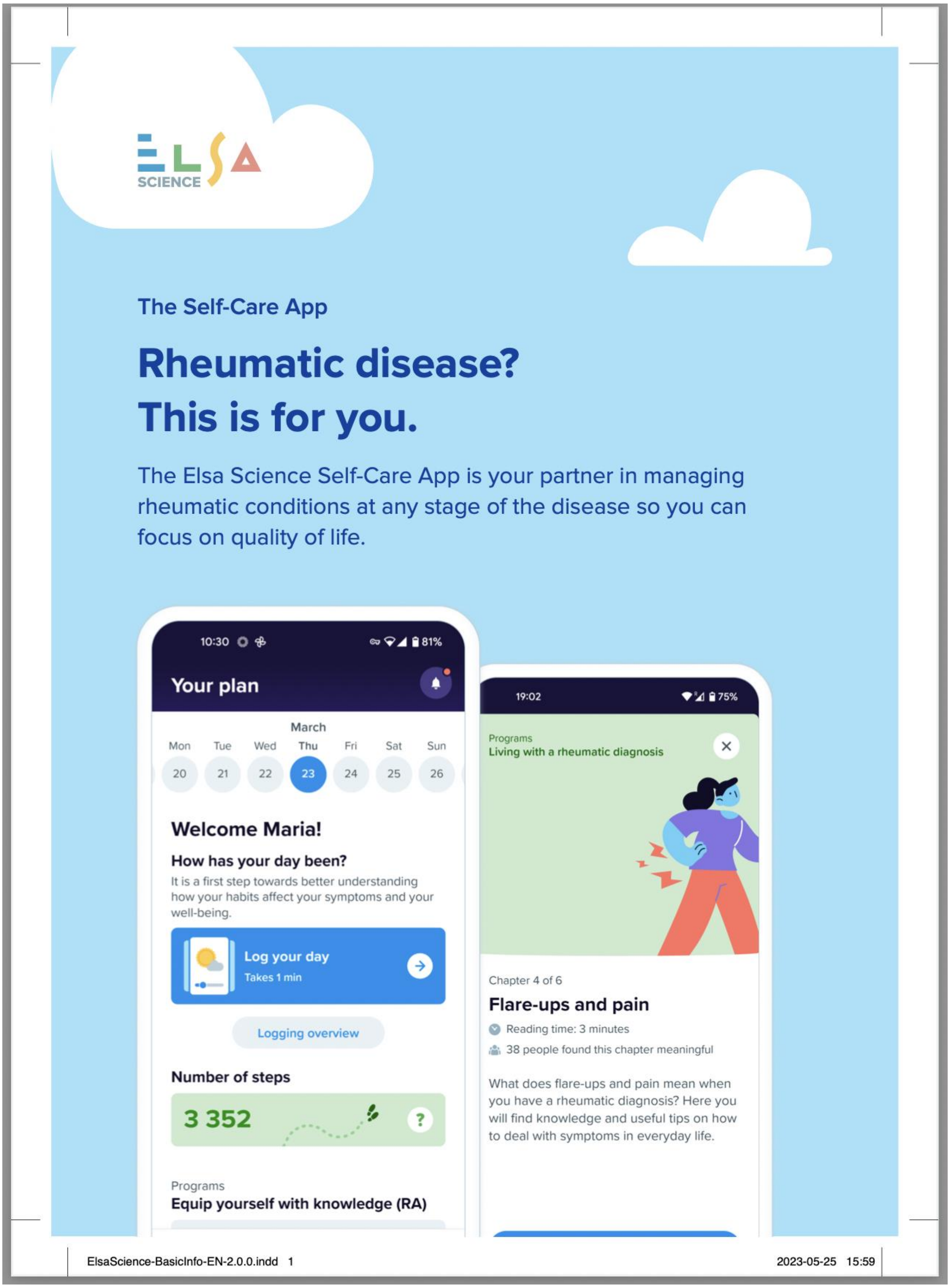
ELSA SCIENCE
The Self-Care App
Rheumatic disease?
This is for you.
The Elsa Science Self-Care App is your partner in managing rheumatic conditions at any stage of the disease so you can focus on quality of life.
10:30
81%
Your plan
March
Mon Tue Wed Thu Fri Sat Sun
20 21 22 23 24 25 26
Welcome Maria!
How has your day been?
It is a first step towards better understanding how your habits affect your symptoms and your well-being.
Log your day
Takes 1 min
Logging overview
Number of steps
3 352
?
Programs
Equip yourself with knowledge (RA)
19:02
75%
Programs
Living with a rheumatic diagnosis
Chapter 4 of 6
Flare-ups and pain
Reading time: 3 minutes
38 people found this chapter meaningful
What does flare-ups and pain mean when you have a rheumatic diagnosis? Here you will find knowledge and useful tips on how to deal with symptoms in everyday life.
ElsaScience-BasicInfo-EN-2.0.0.indd 1
2023-05-25 15:59
