# Supplementary material for: Experiences of individuals with rheumatoid arthritis interacting with health care and the use of a digital self-care application: a qualitative interview study
Source: BMJ Open. 2023 Dec 20;13(12):e072274. doi: 10.1136/bmjopen-2023-072274 (PMC10748980; doi:10.1136/bmjopen-2023-072274)
Supplement: Author's manuscript [file bmjopen-2023-072274.draft_revisions.pdf]

BMJ Open

BMJ Open is committed to open peer review. As part of this commitment we make the peer review history of every article we publish publicly available.

When an article is published we post the peer reviewers' comments and the authors' responses online. We also post the versions of the paper that were used during peer review. These are the versions that the peer review comments apply to.

The versions of the paper that follow are the versions that were submitted during the peer review process. They are not the versions of record or the final published versions. They should not be cited or distributed as the published version of this manuscript.

BMJ Open is an open access journal and the full, final, typeset and author-corrected version of record of the manuscript is available on our site with no access controls, subscription charges or pay-per-view fees (http://bmjopen.bmj.com).

If you have any questions on BMJ Open's open peer review process please email info.bmjopen@bmj.com

BMJ Open

Experiences of people with rheumatoid arthritis interacting with healthcare and the use of a digital self-care application – an interview study

Journal:	*BMJ Open*
Manuscript ID	bmjopen-2023-072274
Article Type:	Original research
Date Submitted by the Author:	27-Jan-2023
Complete List of Authors:	Viberg Johansson, Jennifer; Uppsala University, Department of Public Health and Caring Sciences<br>Blyckert, Hanna; Elsa Science<br>Schölin Bywall, , Karin; School of Health, Care and Social Welfare, Division of Health and Welfare Technology; Uppsala University Department of Public Health and Caring Sciences, Centre for Research Ethics & Bioethics
Keywords:	Rheumatology < INTERNAL MEDICINE, Self Care, Patient Participation

SCHOLARONE™
Manuscripts

I, the Submitting Author has the right to grant and does grant on behalf of all authors of the Work (as defined in the below author licence), an exclusive licence and/or a non-exclusive licence for contributions from authors who are: i) UK Crown employees; ii) where BMJ has agreed a CC-BY licence shall apply, and/or iii) in accordance with the terms applicable for US Federal Government officers or employees acting as part of their official duties; on a worldwide, perpetual, irrevocable, royalty-free basis to BMJ Publishing Group Ltd ("BMJ") its licensees and where the relevant Journal is co-owned by BMJ to the co-owners of the Journal, to publish the Work in this journal and any other BMJ products and to exploit all rights, as set out in our licence.

The Submitting Author accepts and understands that any supply made under these terms is made by BMJ to the Submitting Author unless you are acting as an employee on behalf of your employer or a postgraduate student of an affiliated institution which is paying any applicable article publishing charge ("APC") for Open Access articles. Where the Submitting Author wishes to make the Work available on an Open Access basis (and intends to pay the relevant APC), the terms of reuse of such Open Access shall be governed by a Creative Commons licence – details of these licences and which Creative Commons licence will apply to this Work are set out in our licence referred to above.

Other than as permitted in any relevant BMJ Author's Self Archiving Policies, I confirm this Work has not been accepted for publication elsewhere, is not being considered for publication elsewhere and does not duplicate material already published. I confirm all authors consent to publication of this Work and authorise the granting of this licence.

For peer review only

Journals: BMJ Open

Title: Experiences of people with rheumatoid arthritis interacting with healthcare and the use of a digital self-care application – an interview study

Short title: Experiences of people with rheumatoid arthritis interacting with healthcare

Authors:
Jennifer Viberg Johansson, Hanna Blyckert, Karin Schölin Bywall

Jennifer Viberg Johansson, PhD
Department of Public Health and Caring Sciences, Centre for Research Ethics & Bioethics, Uppsala University, Uppsala, Sweden
jennifer.viberg-johansson@crb.uu.se
+46 18 471 62 88

Hanna Blyckert, BSc
Elsa Science, Stockholm, Sweden
hanna@elsa.science

Karin Schölin Bywall, PhD
School of Health, Care and Social Welfare, Division of Health and Welfare Technology, Mälardalen University, Västerås, Sweden
Karin.scholin.bywall@mdu.se
&
Department of Public Health and Caring Sciences, Centre for Research Ethics & Bioethics, Uppsala University, Uppsala, Sweden

Corresponding author: Jennifer Viberg Johansson

Keywords: self-management, mobile application, patient perspectives, patient preferences, pedagogic program, rheumatoid arthritis

Word account: 8310

For peer review only

Funding

This project was supported by Vinnova, Innovationsfonden and The Research Council of Norway, under the frame of Nordforsk (Grant agreement no. 90825, Project NORA). The funding agreement ensured the authors' independence in designing the study, interpreting the data, and writing and publishing the report.

Competing interests

Karin Schölin Bywall and Jennifer Viberg Johansson have no conflicts of interest to declare. Hanna Blyckert is employed at Elsa Science as a Health educator and user researcher.

Data sharing statement

All data relevant to the study are included in the article.

Ethics approval statement

The study was approved by the Swedish Ethical Review Authority (Dnr: 2021-05431-01). Consent to participate was obtained from all respondents, both oral and written. This work was carried out in accordance with the Declaration of Helsinki and prioritised respondents' privacy and safety. When we requested consent, the respondents were informed that they could withdraw from the study at any time with no explanation, and that ending would not affect the current treatment. Their names were replaced with codes; all personal identifiers were removed so that the persons described, or narratives, were not identifiable.

Contribution Statement

JVJ, HS and KSB conceptualized the study. HB preformed the interviews. JVJ and HB analysed the interviews and interpreted the data. JVJ was the major contributor in writing the manuscript. HS and KSB substantively revised the manuscript. All the authors read and approved the final manuscript.

Acknowledgment

Our greatest gratitude goes out to all the respondents who participated in the interviews. Thank you for your time and for sharing your experiences and opinions so generously.

For peer review only

Abstract

Objectives: Over the last decades, there has been great progress in the improvement of rheumatoid arthritis (RA) treatment. This relates to drug development and the development of guidelines for teamwork and patient self-care, and access to digital tools. This study aimed to explore the experiences of people with RA interacting with healthcare. Another aim was to explore opinions on how a self-care application, with an educational program, "The healthcare encounter", was improving the patient-doctor communication.

Design: Semi-structured interviews were conducted and qualitative content analysis were preformed.

Setting: The potential respondents, people with established, or under investigation of, RA diagnosis, were asked to participate in the study via a digital self-care application.

Participants: Ten interviews were performed after the meeting with the rheumatologist or other healthcare personnel between September 2022 and October 2022. Phrases, sentences, or paragraphs with experiences from healthcare meetings and opinions about the digital program were identified and coded. Codes that reflected similar concepts were grouped; sub-categories were formulated, and categories were connected to their experiences and opinions.

Results: Among our respondents, we found four main experiences interacting with healthcare: the availability of healthcare, whether healthcare includes a holistic approach to tackle health issues, their individual efforts to have a healthier life, and the personal interaction with healthcare. Respondents described that the educational program can be a source of information, which confirms, supports, and creates a sense of control.

Conclusion: The respondents valued being seen and taking part in a dialog when they had prepared themselves (observed symptoms over time and prepared questions). Implementation of digital self-care applications might need to be incorporated in the healthcare setting so that both the patients and the healthcare personnel are on the same page. After all – it takes two to tango.

Strengths and limitations of this study

- Patient perspective on interacting with healthcare was explored through semi-structured interviews.
- Patients' opinions on whether a self-care application was improving the patient-doctor communication was explored.
- This may not be transferable to other disease groups; it indicates how this particular group at this disease stage experiences healthcare
- The invitation to participate in this interview was distributed to potential participants via a mobile application. Patients not using this mobile application were therefore excluded.

For peer review only

Introduction

Rheumatoid arthritis (RA) is an autoimmune, systemic, inflammatory, chronic condition causing joint pain and synovitis.[1] The disease affects around 0.5–1% of the population.[2 3] Synovium happens in the joints due to the malfunction of the immune system. The disease typically affects one particular joint on both sides of the body, like both hands, knees, or ankles. RA also causes degenerative problems in other parts of the body, such as the eyes, heart, and circulatory system and/or lungs.[4] The disease usually develops over weeks and months. However, symptoms can come overnight as well. Many people experience unnatural fatigue, a general feeling of illness, as well as swelling and soreness in the hands and feet, since the disease often attacks small joints first. Morning stiffness for an hour or more is typical. [5]

During the last decade, great progress has been made in the improvement of RA treatment, and the research on treatments for RA is at the forefront of both drug development and the development of guidelines for teamwork and patient self-care.[6] There is still no cure for the disease, but medications can reduce symptoms and help slow the progression of the disease development so that the person affected maintains good function in the joints. Several administration modes and medications can be combined to achieve the best effect on the symptoms. The treatment needs to be adjusted throughout life because of new symptoms, lack of effectiveness, side effects or other components. Besides effective medication, other components of the RA treatment need to be considered, such as education about self-care and self-management, to increase adherence to medication. Communication skills are also important for rheumatologists and other healthcare personnel to promote shared decision-making between patient and the rheumatologist and enhance patient empowerment.[7]

Apart from the physical changes resulting from RA, people also face many psychological challenges like distress and helplessness.[8] They need to cope with a general feeling of being unwell, which can result in decreased ability to participate in daily life, social, and recreational activities. Psychological well-being (including e.g. self-esteem, self-perception, self-worth, body image, relationship with their partner, and limitations in sexual activity) has been shown to correlate negatively with the level of joint tenderness in people with RA.[9]

Besides finding the right medication use and hindering the development of disabilities in the joints, guidelines recommend providing education and tools for handling psychological aspects of the disease.[4] There are recommendations to help patients identify factors and means to manage symptoms like pain, fatigue, depression, anxiety, and sleep problems.[7] Earlier research has emphasised the need for education in self-management skills and counselling to enhance empowerment and improve the emotional dimension of care. Findings support the need for team-based rehabilitation interventions to enhance empowerment in patients with RA.[10 11] Moreover, results from a review of RA patients' self-management support needs show that people with RA have informational, emotional, social, and practical needs. Informational needs can be about how to deal with pain, exercise, and medication. Emotional support can be from their relatives or healthcare professional.[12]

Using mobile technology as a complement in healthcare has increased during the last decade and is now a natural part of healthcare-related services in general,[13 14] as well as for people with RA who require daily self-management of the disease.[6] The shift towards using digital tools has changed healthcare in many ways. RA patients can now inform themselves using trustworthy sources, log their status and self-manage the progression of the disease without

For peer review only

having to be at the hospital. The digital tools come with a promising opportunity for self-management and can be helpful in preparing for interactions with healthcare. Moreover, adding digital tools has been shown to be more effective for treating RA and is therefore also cost-effective.[15] However, comparing the traditional care with the more collaborative care, a more shared expertise is needed between the lived experience of RA and healthcare knowledge. Both collaborative care and self-management education shift focus onto people's own responsibility for their health. Yet, great responsibility remains with the healthcare personnel, who have an active role in communicating, activating, and assisting patients in the self-management of their condition.[16]

The medical evidence, international and national guidelines and legal prerequisites, and digital technologies have been rolled out to enable good care of RA patients. The question is whether the healthcare in Sweden is ready for a more active patient, and whether such interactions between people with RA and the rheumatologist work well in today's healthcare. There is a need to explore how people with RA experience the interaction with healthcare and the use of digital tools to improve self-care and communication.

This study aimed to explore the experiences of people with RA, or under investigation for RA, interacting with healthcare. Another aim was to explore opinions on how helpful a digital self-care application was in improving the patient-doctor communication.

For peer review only

Methods

Design

The study was a qualitative semi-structured interview study.

Respondents and the setting

The potential respondents, people with established, or under investigation of, RA diagnosis, were asked to participate in the study via a digital self-care application called Elsa (https://www.elsa.science/en/). The structure of the study was to first participate in a survey, then perform a specific program in the Elsa app called "The healthcare encounter" and, in conclusion, do a final survey. In the final survey, they had the chance to sign up for a follow-up interview.

The aim of the specific program is to provide basic knowledge to individuals living with a rheumatic disease that can inspire them to make sustainable lifestyle changes and improve their well-being. "The healthcare encounter" program focuses on providing basic knowledge about the treatment, medical options, what to expect from meeting healthcare, and how the individuals can prepare themselves before the meeting. See Box 1.

Box 1.

**The content of the educational program**
- Introduction, background and basic knowledge<br>- The goal of the encounter (treatment goals)<br>- The treatment in general (different medication options)<br>- Long-term collaboration (the healthcare system's role and your role as a patient)<br>- How to prepare (before the encounter)?<br>- How to act during the encounter?<br>- How to follow-up after the encounter?<br>- Checklist

Data collection

Eighteen out of 43 persons declared their interest in participating in this interview study. The respondents were then asked via e-mail to schedule an interview. Not all could be included as they did not have any encounter planned with the healthcare. Ten interviews were performed after the meeting with the rheumatologist or other healthcare personnel between September 2022 and October 2022 by the second author (HB).
The interviews lasted 44 to 63 minutes and were conducted in Swedish via Google Meets or telephone (n=1). We began each interview by asking about their experience of having RA, their treatment and health in general. Thereafter, we asked them to describe their experience before, during and after the healthcare meeting. They were also asked to describe the use of the digital program *The healthcare encounter*, as to whether it was useful to them or if it was missing any information or function. A semi-structured interview guide with open-ended questions[17 18] was developed with all authors; see Table 1.

For peer review only

Table 1. Interview guide used for the semi-structured interviews

Can you please tell me about yourself and your life at the moment (warm-up question)
Before your appointment with healthcare– how did you feel before your visit?<br>Probing questions: Did you prepare in any way. If so, how? Was there anything specific you planned to bring up at the meeting? Did you get any help from the program in your preparation? If so, what? Was there any difference in how you prepared now compared to how you usually prepare yourself?
During the appointment with healthcare– how was the visit?<br>Probing questions: What did you talk about (feeling in general, treatment, side effects, etc.)? What felt good/less good during the meeting? Did you have the opportunity to bring up what you wanted to talk about (treatment options, any side effects, other complaints, what is important to you)? Compared to previous visits, how did you experience this?
After the appointment with healthcare– do you remember how you felt after the visit?<br>Probing questions: Was the meeting as you wanted it to be? Would you like to change anything if you could in retrospect? In retrospect, is there anything that feels unclear after the meeting (missing info, unsure of how to do something, proceed with self-care)? How have you handled the uncertainty?
About the program– what do you think about the program?<br>Probing questions: Was there something that you particularly liked? Something you liked less or even disliked? Is there something you missed or would like more of?

Patient and public involvement

No patient involvement in the interview guide development.

Analysis

The recorded interviews were transcribed verbatim by a professional transcription company. The transcripts were listened to in its entirety to verify the transcriptions. After all the transcripts were read again, meaning units (phrases, sentences, or paragraphs), with experience about healthcare meetings and opinions about the digital program, were identified for further scrutiny. The material contained 344 meaning units in total. Atlas.ti Web[19] and Microsoft Excel (2016) were used to assist in the data management and analysis process. In the next stage of the process, we continued with comparisons of the meaning units, examining their similarities and differences from the perspective of experiences and options of healthcare and the digital program. Open coding of each meaning unit was added, which summed up what was being said in the text. Two of the interviews were coded simultaneously by both authors (JVJ and HB) who jointly discussed what meaning units to identify, interpretations and formulations of codes. The rest of the interviews were coded by JVJ. Codes that reflected a similar concept were grouped together; sub-categories were formulated, and categories were identified[20 21] by JVJ and thereafter discussed thoroughly with HB and KSB; see Table 2. Thematic saturation was reached in relation to the aim of the data collection.

Table 2. Example of the analytical process of the experiences when interacting with healthcare.

Meaning unit	Initial coding	Sub-category	Category
But like [trying to get in touch], you just feel, what the hell, I have no power.	Does not reach care	Accessible healthcare	Availability of healthcare
I have expressed several times to them on the phone, I feel alone. I feel very alone. I think it is hard actually. But then, it's not like I'm lying down and crying about it, but I feel left out.	Feeling alone and left out	To be taken care of	A holistic approach to tackling health issues

For peer review only

He is responsive, he listens, understanding […] it is a pleasure to go and see him. I never feel any stress or pressure when I talk to him. He is very responsive and listens... he always takes what I say seriously.	He is responsive, listens, and explains; he takes what I say seriously.	Met with interest	Personal interaction

Results

In total, 10 interviews (seven females, three males) were conducted with persons with RA, or under investigation (n=1 under investigation with symptoms two years back in time). Disease duration ranged from one to three years, with one exception, where the person received a diagnosis 12 years ago. The respondents were aged 45–76 years (mean age 56.7) and from different demographic locations in Sweden. All respondents described their experience of being diagnosed with RA as a challenge. Those who were still unsure of what their symptoms indicated expressed frustration about not knowing or understanding the body's signals. They had tried various healthcare services that might be helpful, and also medications that might work. Having problems linked to other diseases was also described as a challenge. Consequently, they described having to choose which health issue to alleviate first. Accepting the disease was experienced as both a challenge and a necessity. What was also described as a challenge, and a necessity, was finding a balance in life between activity and recovery, work and leisure, and physical activity and rest.

The next section describes the respondents' experiences of interacting with healthcare. The qualitative content analysis revealed four main categories. Thereafter, 12 sub-categories were used to classify the discussions. The categories were: 1) Availability of healthcare, 2) A holistic approach to tackling health issues, 3) Individual effort, and 4) Personal interaction. An overview of the categories and subcategories is presented in Table 3. Below, these categories and sub-categories will be described and illustrated by quotes.

For peer review only

Table 3. The categories and sub-categories of respondents' experiences interacting with healthcare.

**Category**	**Sub-category**
Availability of healthcare	To feel prioritised
	Accessible healthcare
	Healthcare that confirms
A holistic approach to tackling health issues	To navigate in healthcare
	To be taken care of
Individual effort	Taking care of myself
	Opportunity to equip myself
Personal interaction	To be seen
	Met with interest
	Gain self-efficacy
	Have a dialogue
	Met with competence

Availability of healthcare

The category *Availability of healthcare* was expressed in three different ways: *To feel prioritised*, *Accessible healthcare*, and *Healthcare that confirms*.

To feel prioritised

This sub-category is about the experience of feeling that there is enough time for patients and healthcare personnel to discuss symptoms, treatment options, and whether resources are invested in helping this group. Some respondents described they felt prioritised and had a great team around them and great support from different healthcare personnel. Some respondents expressed a feeling of not being prioritised in situations when they met healthcare. They stated that the care meeting was too brief, so that one simply did not have time to discuss things related to the disease, besides medication; moreover, they also did not have the possibility to ask follow-up questions. The time constraint was perceived as preventing a personal meeting. They expressed a feeling of not being treated like a human being; it was more like an assembly line.

> *Well, I would have liked his [the rheumatologist] clinical part first[…] goes through the joints and any blood tests and explains what the result means, so that you might learn something yourself. So you don't have to keep asking more times. And I wish him to ask, 'how does everyday life work for you?' I still have not been asked that question since 2019. (Respondent 3)*

Some respondents suggested the benefit of having more frequent meetings early on in the process, when more issues arise and there have concerns. The possibility of having more meetings, if needed, gives the impression that someone is taking care of you. Being met by healthcare personnel who do not have knowledge of rheumatic disease can make you feel insecure, resulting in a feeling that this patient group is not prioritised.

> *..then I felt some insecurity. If it were someone who had the actual expertise, then they may have asked other follow-up questions. The person was very nice, but it felt a little unsafe. It felt like, who is taking care of me? It's not her fault, of course, but that's how I felt...(Respondent 7)*

Accessible care

Having healthcare that is accessible is highly appreciated and valuable for the respondents. Some expressed that it was easy to make contact via phone or messages via the digital platform used in Sweden (1177). When problems with symptoms or side effects occur, fast response, action, and reply were appreciated, giving a calming feeling. Having regular meetings scheduled also led to feeling safe.

> *I think it [meeting the rheumatologist] feels safe. I think it feels great. So, I'm happy to continue with that. (Respondent 5)*

However, not all respondents experienced this. Some felt it was difficult to reach healthcare via phone or messages. One described it as a whispering game; you call and tell a healthcare personnel the problem and that message is forwarded to the rheumatologist, who then sends a message back via a third person. These difficulties in reaching healthcare are perceived as unsafe care, resulting in the feeling of being exposed to danger.

> *... and I know that care is generally heavily burdened, so without talking badly about an individual. But like, you just feel, what the hell, I have no power.(Respondent 7)*

For peer review only

Healthcare that confirms

The fact that the care provides confirmation is also highly appreciated and valuable to the respondents. Some described that the rheumatology clinic does a fantastic job and that you, as a patient, get quick answers if you have concerns or experience practical obstacles with medications. They also stated they receive a good follow-up of blood test results. The participants who experienced collaboration regarding the treatment of RA expressed that the various professions had good accessibility and followed up on their work, and it was clear how to reach out to them when a problem occurred.

> *I have to say that I am very impressed with the rheumatologist at the hospital [...] and I do that [send a message] and it can take within an hour then I've got a response that either the nurse has answered, or she says that, "I've passed it on to your doctor" and then in the afternoon, I get an answer. So, this is how it has been for these four years, fantastic. (Respondent 5)*

However, for other respondents, it can take a long time to get confirmation of test results. Sometimes it is not until the next appointment or if they have indicated they are not doing well.

A holistic approach for tackling health issues

The category *A holistic approach for tackling health issues* was expressed in two different ways: *To navigate in healthcare* and *To be taken care of.*

To navigate in healthcare

Knowing where to turn and receiving help was perceived as important by the respondents. Some described they had access to direct numbers if they had a flare-up. Some expressed they had a good routine for sending a message to the rheumatologist, who replied to them quickly. Others expressed difficulties in that they were sent around in healthcare: Primary care to different specialists and then back to primary care. For respondents who had many different ailments, where it was difficult to know what was what in the illness, they talked about the difficulties in finding the right help. They described a long journey to finally receive help for their symptoms and a diagnosis. They claimed it was difficult to understand the course of healthcare; moreover, they were frustrated that different healthcare units did not communicate with each other. Some respondents talked about how they had to be the one who coordinated the care. This was perceived as tiring and difficult. Finding the right care can be an obstacle and take a lot of endurance. When it was not clear what treatment works and what symptoms to prioritise, the respondents thought it took a lot of effort and energy. Respondents who had many symptoms and also experienced other diseases at the same time expressed great difficulty in finding the right care in complex situations. One respondent considered changing to a bigger hospital to receive better care, having to consider a longer journey.

> *[…] why should I change [health care unit] then? …as I understand it, it is a bigger hospital. After all, there is a larger clinic and more doctors who are specialised. Often, when I call my specialist's office, it's... well, it's the diabetes nurses I get to talk to, it's like... I do not know what it is like in there [at the bigger hospital], but it sounded like it could be different. So I*

For peer review only

> *said, of course, I want to change because I want to be where I get the best help; of course...(Respondent 7)*

Some felt they have been sent around to different units to have their concerns heard. It can also be difficult as a patient to know where to go with the problem that is occurring: is it the nurse, the physiotherapist, the occupational therapist, the curator, or the rheumatologist?

> *[…] it is not crystal clear, where to go in healthcare. And I'm a migraine person. A lot of small problems. But I'm a very stubborn person, and I don't give up. I fight.*

> *[…]you are sent around a lot between different healthcare units, depending on what problems arise. And there is no connecting link. One part of healthcare does not know what the other part is doing. (Respondent 6)*

To be taken care of

All respondents appreciated having healthcare personnel who conveyed a feeling that one is being taken care of. Some respondents expressed great gratitude and were impressed by the teamwork they had experienced. They felt getting the right support gave a feeling of being taken care of, trustworthiness in the care, and the feeling of having support if difficult times were to come. A noble example, told by one of the respondents, was when the healthcare profession helped to refer her onward, instead of letting the patient seek help again on her own. The respondents experienced great teamwork and expressed pride in their care unit, stating that the staff have a nice and welcoming attitude, and the feeling of being looked after was strong.

> *…then he [the rheumatologist] had been talking to the gastroenterologist and discussed with them and put together an action plan with them. (Respondent 6)*

Some respondents had the opposite experience: frustration at being sent around or not getting good follow-up on their symptoms, blood tests or medication. They expressed that it caused concern and created incredible insecurity as to whether someone has control over the situation. The respondents asked themselves if they did not to hear from the healthcare system, what does it mean: that they do not care, that they have forgotten about me, or that the test results are without remark? It said that not hearing from healthcare resulted in a feeling that no one watch over you.

Respondents felt that you needed to time it, so you were sick enough at the meeting to receive the right help. Being well at the time of the appointment resulted in not getting the right help. Because symptoms come and go, some respondents found it difficult to find an appointment time when they were really bothered; consequently, they did not feel they were being taken care of.

> *And maybe also some form of follow-up. I mean, if he doesn't find an inflammation now, I might have it in a month or a week. That they sort of had some kind of... yes, but "if you get an inflammation, get in touch and come here and then show us and then we'll get... and then we'll take new measures". Instead of just ending the meeting and then I am sent back to primary care. (Respondent 8)*

For peer review only

Individual effort

The category *Individual effort* was expressed in two different ways: *Taking care of myself* and having the *Opportunity to equip myself.*

Taking care of myself

All respondents expressed they try to adapt to life in different ways and undertake different attempts at self-care: they have adjusted their diet, tried different forms of exercise and training, and tried to find a balance between rest and activity. The respondents described how they prepared themselves in different ways before the visit for the conversation with the rheumatologist. In addition to taking notes on how they feel – either with pen and paper or via digital tools – they sought information in different ways to equip themselves with knowledge. Some stated that equipping themselves with new knowledge was for their own sake. Others stated it was also to facilitate healthcare, so they can be involved in the 'detective work' of finding effective treatment. Moreover, they try to understand and interpret their own test results on the health and medical care's digital platform (name 1177) before the meeting. To write down questions and register symptoms and how they have felt over a longer period were common actions. This was, for some, useful as a tool to reflect on their health, understand the reasons for the symptoms, how they come and go, but also to remember the ups and downs of their symptoms.

> *I had a long list I had written before the phone meeting, where I tried to think "how long have I been in pain" and "where do I have pain" and "how do I react" and "what medicines have I taken" and so on. So that you don't forget anything when you talk to the physician. (Respondent 6)*

Some expressed frustration that they must know a lot themselves to get help, that you have to stand up for yourself to get good help and that it feels like one has to be healthy to be able to be sick.

> *I don't think it's okay, I told her [...] all the side effects. Then it was like "well, okay, let's try something else". I myself have had to argue a lot to change my medication… (Respondent 5)*

Some participants expressed a positive feeling about taking part in research in the field of RA. They described it gave a nice feeling of being able to give back to healthcare for all the good things they have received.

Opportunity to equip myself

There was great variation in the extent to which our respondents had the opportunity to be educated regarding their disease. Some had four days of education, some had one day or even half day, and some did not receive any education from their rheumatology clinic. To receive education about the disease, medication, exercise, lifestyle habits, and how to find a balance in life was much appreciated by the respondents who experienced that. They felt it made them feel safe and satisfied even though life has changed with the diagnosis. Education was perceived to empower them and made it possible to take care of themselves.

> *... the introductory education... I mean, four full days. And so, they have it regularly [...] with everyone who is newly diagnosed, where they go through [everything]. And they also went through which medicines are available, and said "and we start here" [...] they were very clear that we*

For peer review only

start here and then you have a period where you test it and if it doesn't work, then we move on to something else. So they informed me about the whole process. (Respondent 6)

Those who did not receive education from the rheumatology clinic expressed a desire for it and hoped and even expected healthcare to provide it. Some described with surprise and disappointment that they had not received it. Understanding one's illness is deemed important; one wants to understand and take power over one's life. Specific aspects for which they wanted more knowledge were medical treatment, side effects, diet suggestions, the diagnosis, and what to expect from life.

…training and such, they talk about that and what is good. That you shouldn't smoke and like... yes, and so on. But diet, in particular, seems to be taboo. (Respondent 8)

Personal interaction

Finally, our analysis revealed experiences related to personal interaction, and how the healthcare professionals' attitudes made respondents feel. This is the most comprehensive category regarding the participants' narratives. This category was expressed in five different ways: *To be seen*, *Met with interest*, *Have a dialogue*, *Gain self-efficacy*, and *Met with competence*.

To be seen

There was a strong emphasis on the appreciation of being seen, namely that professionals would meet the patient with empathy. Respondents wanted to feel as though they were believed, not perceived as whiny, and to gain some hope in difficult situations. They wanted to be seen as a whole individual and talked to as an equal, and that personnel did not look down on them. When the doctor takes the time and listens, the feeling of being seen as a person increases.

Very professional, very calm, nice and she gave me hope. (Respondent 5)

Some stated they felt stupid when interacting with the rheumatologist. They emphasised the importance of including the patient's perspective, or at least having a nice response. Not being listened to resulted in a despondent feeling as if "it doesn't matter what I do". It also led to a cold, not human, robot-like feeling of the relationship. Respondents stated that if one has prepared and written down how they have felt lately, then it is important that this information is not overlooked. Focusing only on medicine and side effects, and not any other aspects of the disease, was not viewed positively. In such cases, respondents felt they were not seen.

… they don't know me. I've met him once, and yet, they somehow assume that… you need to bite the bullet. It's a bit like that. And the first time ever when I was at the health centre, when my fingers hurt... and then I had really red knuckles. They were really inflamed. Then the medical centre doctor told me that you have to learn to live with that, you just have to bite the bullet. (Respondent 8)

Met with interest

Besides being seen, the feeling that the rheumatologist is interested in the patient's story and takes time to answer is valuable. The professional approach was important to patients, where

For peer review only

the attitudes and responses of healthcare personnel can help patients feel respected. Being on time, listening and meeting the patient's expressed needs, gave a feeling of respect. If the healthcare does not call back as they promised or does not write down the patient's medical records correctly, or not at all, it is a disappointment. This was viewed as the health care not being interested in them, that they are just one on a product line. Nevertheless, the fact that the rheumatologist is well read and prioritises the right things to discuss at the meeting were perceived very positively, making the patient feel as though he or she is seen with interest. Respondents expressed a positive attitude when they were seen as a resource; the doctor is the medical expert, while the patient is the expert on themselves. They felt that for the encounter to be as good as it could be, they both needed to share their expert knowledge with one another.

> *...since she is so calm and takes her time and listens to me, I don't have to stress through them [the questions] or skip any. I may have twenty questions, of which five are very important, and fifteen are... if we have time, I will ask them. Then I can get answers to something that I have wondered for years. (Respondent 4)*

Some respondents with bad experience with healthcare described they feel that for the rheumatologist, they are just a body to be examined. They felt they are not allowed to talk about where they have pain and for which symptoms they would like help to alleviate. One participant felt that her own illness story was uninteresting to the rheumatologist. Another respondent expressed she sometimes feels that she needs to lie about her current pain during a healthcare visit in order to get help, even if the flare-up of pain this time has just passed. This is because she is worried that her complaints will not be taken seriously just because she is feeling a little better at the moment. The meeting with the doctor is a cross-section of the person's life and that is frustrating to deal with. There is a wish to understand on what grounds the physician makes his or her medical decision. The respondents expressed a wish that the lived experience of the symptoms and the side effects can be used in the treatment discussion.

> *... the physician says that the physician's task is to fix the medicine and I take care of me, what is best for me. But then I have to tell the physician how I feel when I do certain things. But no one ever cares about that, it's just, what does the medicine say, only the blood measurements count, how does it feel in the finger joints, period (Respondent 6).*

Have a dialogue

The respondents appreciated having a good dialog with the rheumatologist; being able to reason with the rheumatologist about medical treatments, side effects and finding a balance between things in life were perceived as valuable. Moreover, the respondents appreciated the rheumatologist taking advantage of the preparation done by the patient before the meeting. Being able to reason about the amount of medicine was also appreciated.

> *I'm a bit fussy, I don't want to take tablets that much, so I stopped after a while, not with Benepali, because I understand that I need it. But these painkillers [...] but now for a period, it hasn't been good and then my doctor said yesterday "one more tablet isn't dangerous and it's not much" [...]. So then, I thought, okay, now I have to listen to her. It might have*

For peer review only

something to do with the fact that I may not have fully accepted this, I thought last night, because I'm so fussy and don't want to take the medicine. (Respondent 5)

For those who did not have the opportunity to have a good dialog, they expressed sadness or frustration about what was missing for them. They all described they had heard that other patients had received better care. When a good dialog was not present, the respondents felt they did not trust the rheumatologist, and either wanted to change the contact-person or go their own way when it comes to medication choices or lifestyle changes. For some, it was not clear whether it was ok to be critical or even change their rheumatologist if the communication does not work out well; it might result in bad consequences.

So, actually, I would like to change my physician. Because there is another doctor there too who helped me at some point when I had a pain in my joints in the palm. He was very nice, accommodating and calm and such. So actually, I want him. But then you don't know if you dare to change the physician like that, because then [...] what if I get the evil eye […]. So, I feel, it's better I go there to those meetings with him, and he does what he has to do, then I google and learn by myself. (Respondent 3)

There was a feeling of resignation among those who prepared but were not listened to. Even though they prepared well (what to ask and how they have felt during the past month), the rheumatologist did not take notice of that: they shared that the doctor did not even look them in their eyes; wasn't interested in the patient's notes or questions; and answered using difficult language. Instead, the rheumatologist went on with a clinical test and made some comment about the medicine. These respondents felt they were being ignored and could instead be a valuable resource.

[…] even though I had kept a log for a very long time, there was nothing that he [the rheumatologist] cared about when I was there. So, he looked there and then, the day I was there. "Are you in pain here, yes. Are you not in pain here, no". And then it was fine with that. (Respondent 8)

Gain self-efficacy

Something that was expressed by all respondents was the need for encouragement and hope for the future. Healthcare was perceived as an important component in the respondents' attempt to find a functional life. It is important to be able to express yourself and be taken seriously; then, there is a feeling of being competent in dealing with the new situation. For some, meeting healthcare can often create a feeling that one has become old and dying. Getting help to set new goals and sub goals was perceived as helpful; it helps to believe in the future.

… I have thought about it quite a lot and that helps me, that I need to have a goal. Not a dream, a goal. Everyone has dreams, but then you can pick certain dreams and say that is a dream, and this is my new goal. The goal for me is to get back on the police motorcycle. And being able to function and not have to think about how I'm paddling a kayak with my wrist or how I'm holding the weight or the dumbbell, or whatever it is. But I should just be able to be, as I was before. (Respondent 3)

For peer review only

Met with competence

If the rheumatologist is knowledgeable and competent, it infuses confidence in the patient. Some respondents are impressed by the rheumatologist's skills in balancing the trade-off between deploying different medications at different times. The respondents felt they get all the answers they need to remain calm and reassured; and following up on any side effects or symptoms was appreciated.

> *...questions about the medicine to the rheumatologist... she is good at explaining (Respondent 4)*

Two respondents felt they received better care if they were prepared and knew about things themselves. Some respondents, however, were disappointed as they did not receive answers to their questions and felt the rheumatologist was only guessing when responding to them, resulting in them feeling resigned.

> *No, but he seems not to care; it feels so stiff when I talk to him. I had to remind him [...]. Well, he's not well-read and that's what annoys me so much when he calls. I know in my heart that he is not well-read. (Respondent 10)*

Opinions about a digital self-care application

A further aim of this study was to explore opinions on how helpful a digital self-care application with the educational program was to improve respondents' ability to express themselves in the patient-doctor communication. They expressed that a digital self-care application can be a *Source of information*, and help to *Confirm*, *Support*, and create a *Sense of control*.

Source of information

Many respondents appreciated the application as a trustworthy source of information with a good spirit.

> *I think it is great [...] easy to understand and at a good level. And it's good to be able to go back and remind myself. (Respondent 9)*

> *So, I google... being critical of sources, it's not that easy as a consumer. This [the application] feels very serious and here they have really talked to people who [are knowledgeable and experienced] ... that's the image I have, anyway. (Respondent 7)*

They valued being able to learn more about what to expect with the disease, to learn about the medication, different healthcare personnel's responsibilities, how to inform relatives, and what activities to engage in to feel better. Learning from other's experiences was also appreciated. One expressed that the app could be useful even if one was not yet diagnosed.

However, two respondents expressed that the information was not a surprise and that it confirmed what they already knew. One respondent believed the content did not always have to be so gentle and nice all the time – life is hard.

Confirm

The respondents claimed that using a digital self-care application creates the feeling of being seen, that someone is carrying and confirming one's experiences. By reading what others have been through, your own experience is confirmed, and you become calm.

> *Other people's stories in the app, […] I just… Yeah, but damn. That's me! (Respondent 4)*

They expressed a positive attitude when someone answered their question via e-mail or the chat function, that someone was replying to their questions and concerns regarding the progression of the disease. It was also mentioned that it is good to digest information and to ask questions at one's own pace, not being rushed or stressed that it is now or never you get the chance to express your concerns. However, one respondent stated that the application's content was not reflecting that person's reality; the reality of how well healthcare works is not as good as described in the application; the app description of healthcare was "too good to be true".

> *[...] the description [in the application] where the doctor looks at the clinical aspects and then I help explain how I feel and how I experience everyday life and what works for me and what doesn't work. Yes, but it was something that I got hooked on and immediately thought yes, but I haven't had the chance to experience that yet. Such a doctor's visit. (Respondent 3)*

Support

The digital self-care application was perceived as a good tool to help prepare before the meeting with healthcare. It also strengthened one's self-esteem before meeting with healthcare.

> *So yeah, I thought it [the content in the application] was... it helped me believe in myself. (Respondent 8)*

After the respondents have gone through the program, they are given a list of good things to prepare for the meeting with the healthcare setting. This list was found to be very helpful to read and think about before the meeting. For the patient, there can be so many thoughts and feelings (e.g. fear, frustration, joy) so it can be difficult to focus on the most important parts. Therefore, they viewed the checklist as a good tool to not forget what is important and help to not lose focus. It also gave the confirmation that it is good to be prepared. To be able to look back on the personal log to remind themselves what had happened over the last period was also appreciated: that was perceived as a good memory support tool. One respondent commented it would be even better if the digital self-care application could invite or push for healthy lifestyle choices. Another suggestion for the application was to include personal goals and help to set interim goals. The function of logging symptoms was also viewed as a good support or basis for reasoning with the rheumatologist. Furthermore, some wished that healthcare had access to their logging data to be able to have better conversations over the previous period and discuss treatment options and outcomes. However, some thought that being constantly reminded of one's illness via the log or reading about all the negative things about the disease can weigh one down.

> *…there is a risk that going on and on like that [with reading and log symptoms every day]... that you dig into your illnesses, and I don't think that feels very good. (Respondent 2)*

For peer review only - http://bmjopen.bmj.com/site/about/guidelines.xhtml

There was also a positive attitude towards including information and educational material to- and by the relatives. It can be difficult for someone affected by symptoms to share that without sounding negative and as if they are complaining. It was stated that information directed to the relatives could ease the burden and facilitate good relations in the family.

Create a sense of control

The respondents thought the app was fun, helpful, and a way of taking care of oneself when filling in the daily log. For some, it gave them time to reflect on how they feel and how the symptoms changed over time. Some thought it would be helpful to log long-term pain, diet, physical activity, general mood and sleeping pattern. They also expressed a need to be more specific in describing the exact location of the pain (e.g. where in the hand). Moreover, they suggested making a note of the possible reason for the pain by typing the activity performed before the pain occurred. On the other hand, some respondents said they forgot or felt they did not want to log their symptoms when everything was fine.

> *I usually go in and use it [logging symptoms], although it's become a bit more sporadic now the last... well, last month and so. Because I have felt better and then when I feel better, I kind of forget to fill it in somehow. (Respondent 9)*

Some respondents pointed out that it was a challenge to log in information when you have symptoms that are not included in the app, for example, neck pain or migraine. Another challenge was distinguishing between what is a symptom due to the illness, ordinary fatigue, or pain from another cause. Respondents thought it was difficult to be objective. Many of them wanted to enter in even more symptoms on the daily log, related to multiple illnesses to understand why the pain arises, to unravel the mystery in order to find good coping strategies and minimise their symptoms; like performing detective work.

For peer review only

Discussion

This study aimed to explore the experiences of people with RA, or under investigation for RA, interacting with healthcare. People with RA are a well-studied group when it comes to the exploration of clinical outcomes of disease-modifying antirheumatic drugs (DMARDs) and patients' views on those clinical outcomes.[22 23] This study brings new aspects to this perspective by asking people with RA about their experience of interacting with healthcare. It is well-known that making shared treatment decisions plays an important role for persons with RA to improve clinical outcomes and their adherence to treatment in the way the DMARD is prescribed.[24]

The main finding of this study was that our respondents valued time and dialog with the rheumatologist. They appreciated and felt it was useful and necessary that their own lived experience of the disease was considered when evaluating symptom relief and discussing further treatment. According to the EULAR recommendations, discussions to introduce the patient perspective could benefit both the patient and healthcare by optimising self-management and treatment adherence.[25]

Besides the physical changes that are inevitable with RA as well as the medical challenges, people with RA face many psychological challenges like distress and helplessness.[8] Our respondents expressed the need to accept the new situation. They also requested help from healthcare to do that. We interpreted that those respondents who had a good experience of teamwork, with several healthcare personnel included in the treatment, had a calmer approach to themselves. We also interpret that if the patients know what to expect from the meeting, when the next meeting is and who to turn to when symptoms increase, it gives them a calmness, which is beneficial for the disease activity overall as well.

Some respondents also appreciated getting help to inform relatives about their diagnosis. It is known that people with RA become more dependent on relationships and their role might change in their relationships.[26] Additionally, they need to cope with fatigue and a feeling of being unwell, which can result in decreased ability to participate in daily life, social and recreational activities. Having mental support from relatives and friends, and the backup knowledge from testimonies using a digital tool, can ease the stress of not feeling alone in the situation.

Given that psychological well-being has been shown to correlate negatively with the level of joint tenderness in people with RA,[9] a more holistic approach is needed. The respondents in this study expressed both positive and negative experiences with regard to including well-being issues in the meeting with the rheumatologist. Common reactions to the diagnosis of RA include feeling overwhelmed and that life has taken a new, unexpected, and unwanted turn. Finding out that you have a lifelong chronic disease can make you sad, create anxiety and fearful for the future,[27] thus perhaps requiring appropriate professional support. There is a risk that, once diagnosed, the immediate focus becomes finding the right drug therapy to reduce inflammation and pain. Finding the right drug is challenging and can take months. After testing a drug, the next follow-up appointment primarily focuses on the efficacy and monitoring of drug therapy.[7] Therefore, it is important to meet those psychological needs as well, and not only look at the joints, inflammation, test results and X-rays. Some of our

For peer review only

respondents expressed frustration at not being seen as an individual, just a body that needs to be examined. This is not in line with the recommendations[25] and should not occur.

Earlier studies show the need for emotional support and guidance on how to live with RA.[28 29] In addition, guidelines recommend providing education and tools for handling psychological aspects of the disease. Another recommendation is to help patients identify factors and means to manage symptoms like pain, fatigue, depression, anxiety, and sleep problems.[7] Previous research has emphasised the need for education in self-management skills and counselling to enhance empowerment and improve the emotional dimension of care. Findings support the need for team-based rehabilitation interventions to enhance empowerment in patients with RA.[10 11] Several psychological interventions have been carried out in order to address these issues of emotional support.[30 31] One study tested having newly diagnosed persons with RA at the clinic meet a rheumatology nurse specialist and talking about the new diagnosis and situation to explore the patients' needs. The appointment gave the patients the opportunity to explore their emotions (allowing them to express their fears and anxieties) and their beliefs about RA, discovering useful coping strategies. The data from this study suggest that nurse-led clinical meetings provide time and space for patients to discuss feelings associated with their new diagnosis of RA, which is appreciated by patients.[27] Moreover, a review of support needs for self-management from RA patients presented that people with RA have informational, emotional, social and practical needs.[12] Informational needs can be about how to deal with pain, exercise and medication. Some respondents in our study were disappointed with the treatment from the rheumatologist, as the rheumatologist did not deal with other aspects of the disease. Could it be that the division of roles is unclear for our respondents and not well described to them? It might be helpful to clarify the role of different professions to the patients and discuss the expectations.

Another aim of this study was to explore opinions on the benefit of a digital self-care application in improving patient-doctor communication. Finding the most suitable treatment for persons with RA is challenging for both patients and healthcare personnel because of the chronic nature of the disease. Self-management using digital tools opens a new treatment era with the potential to better meet the needs and preferences of persons with RA, improve overall health outcomes, and increase satisfaction with their interaction with healthcare personnel.[32] As our respondents expressed, living with RA can be different from day to day depending on disease symptoms or treatment side effects. The individual with RA carries the burden of managing their daily life, which may also include coping with pain, fatigue and medications, etc. In this regard, most of the activities to promote health in persons with RA occur outside of the rheumatologist's office.

Our respondents appreciated when the healthcare personnel took their observations into account when following up on disease activity. However, not all had a good experience of that, which was permeated with disappointment and surprise. Therefore, we would like to argue that strengthening self-management by using a digital tool log on what happens between visiting healthcare should be promoted by the healthcare, not by the individual person with RA. As for the healthcare perspective, digital tools for self-management may increase the efficiency and time spent during visits, improving relationships with patients, and using more precise data to guide treatment decisions.[6] In addition, regarding the meeting with the rheumatologist and patients' wish to be seen, a simple question about 'how has it

For peer review only

been for you lately' might change the perception of the meeting. Although none of the respondents expressed they had received bad medical treatment, some respondents did experience a bad patient-physician interaction. This can be improved by very small changes, e.g. including interested responses on the patient's own experience and reflections could increase the quality of the meeting.

Different digital tools could be a solution to improve access to care in parts of the country that do not have teamwork for this patient group. It can also improve timeliness of care, addressing urgent health issues quickly. It could also improve efficacy of care by supporting improved outcomes with closer follow-up. That could include web-based education, videoconference, telephonically, or electronic messaging. This has been used successfully in oncology service and treatment,[33] but has also increased in healthcare overall during the COVID-19 pandemic.[34] Whether virtual-care modalities for RA can replace in-person rheumatologist visits, while maintaining the comparable quality of care, has been investigated in a systematic review. They summarised the limited existing evidence regarding the impact of virtual rheumatology care in patients with RA on patient disease activity and patient experience/satisfaction with care. They found no difference in observed outcomes between virtual care delivered by a rheumatologist and by a rheumatology nurse. However, virtual care was found to have additional benefits for improved treatment adherence, maintenance of functional status, and quality of life.[35] However, it is critical that those digital tools are evidence-based and focus on self-management interventions that are developed by healthcare providers and persons with RA in order to support and empower individuals.[36]

Conclusion

What we know is that technology is here to stay in our society and in healthcare settings. Thus, more thinking needs to be done on how to best deploy digital tools effectively; specifically, how to balance digital meetings, physical meetings, patients' own preparation and what healthcare can provide regarding treatment. Our respondents' feelings about not being seen and being part of a dialog when they have logged their symptoms and prepared themselves might be due to an implementation gap between healthcare and the patients. The next step might be to implement digital tools in the healthcare setting, so both the patient and that healthcare personnel are on the same page. After all – it takes two to tango.

For peer review only

References

1. Conaghan PG, Green MJ, Emery P. Established rheumatoid arthritis. *Best Practice & Research Clinical Rheumatology* 1999;13(4):561-75. doi: https://doi.org/10.1053/berh.1999.0046
2. Smolen JS, Aletaha D, McInnes IB. Rheumatoid arthritis. *Lancet* 2016;388(10055):2023-38. doi: 10.1016/s0140-6736(16)30173-8 [published Online First: 20160503]
3. Incidence and prevalence of rheumatoid arthritis, based on the 1987 American College of Rheumatology criteria: a systematic review. Seminars in arthritis and rheumatism; 2006. Elsevier.
4. Aletaha D, Neogi T, Silman AJ, et al. 2010 Rheumatoid arthritis classification criteria: an American College of Rheumatology/European League Against Rheumatism collaborative initiative. *Arthritis Rheum* 2010;62(9):2569-81. doi: 10.1002/art.27584
5. Majithia V, Geraci SA. Rheumatoid Arthritis: Diagnosis and Management. *The American Journal of Medicine* 2007;120(11):936-39. doi: https://doi.org/10.1016/j.amjmed.2007.04.005
6. Mollard E, Michaud K. Self-Management of Rheumatoid Arthritis: Mobile Applications. *Current Rheumatology Reports* 2020;23(1):2. doi: 10.1007/s11926-020-00968-7
7. Smolen JS, Landewé RBM, Bijlsma JWJ, et al. EULAR recommendations for the management of rheumatoid arthritis with synthetic and biological disease-modifying antirheumatic drugs: 2019 update. *J Annals of the Rheumatic Diseases* 2020;79(6):685-99. doi: 10.1136/annrheumdis-2019-216655
8. Treharne GJ, Lyons AC, Booth DA, et al. Reactions to disability in patients with early versus established rheumatoid arthritis. *Scand J Rheumatol* 2004;33(1):30-38. doi: 10.1080/03009740310004685
9. Gettings L. Psychological well-being in rheumatoid arthritis: a review of the literature. *Musculoskeletal Care* 2010;8(2):99-106. doi: https://doi.org/10.1002/msc.171
10. Larsson I, Bremander A, Andersson M. Patient Empowerment and Associations with Disease Activity and Pain-Related and Lifestyle Factors in Patients With Rheumatoid Arthritis. *ACR Open Rheumatology* 2021;3(12):842-49. doi: https://doi.org/10.1002/acr2.11341
11. Donnelly S, Manning M, Mannan H, et al. Renegotiating dimensions of the self: A systematic review and qualitative evidence synthesis of the lived experience of self-managing rheumatoid arthritis. *Health Expect* 2020;23(6):1388-411. doi: https://doi.org/10.1111/hex.13122
12. Zuidema RM, Repping-Wuts H, Evers AWM, et al. What do we know about rheumatoid arthritis patients' support needs for self-management? A scoping review. *International Journal of Nursing Studies* 2015;52(10):1617-24. doi: https://doi.org/10.1016/j.ijnurstu.2015.05.008
13. Perry B, Geoghegan C, Lin L, et al. Patient preferences for using mobile technologies in clinical trials. *Contemporary Clinical Trials Communications* 2019;15:100399. doi: https://doi.org/10.1016/j.conctc.2019.100399
14. Nikiphorou E, Santos EJF, Marques A, et al. 2021 EULAR recommendations for the implementation of self-management strategies in patients with inflammatory arthritis. *J Annals of the Rheumatic Diseases* 2021;80(10):1278-85. doi: 10.1136/annrheumdis-2021-220249
15. Bodenheimer T, Lorig K, Holman H, et al. Patient Self-management of Chronic Disease in Primary Care. *JAMA* 2002;288(19):2469-75. doi: 10.1001/jama.288.19.2469 %J JAMA
16. Von Korff M, Tiemens B. Individualized stepped care of chronic illness. *The Western journal of medicine* 2000;172(2):133-7. doi: 10.1136/ewjm.172.2.133 [published Online First: 2000/02/29]

For peer review only

17. Kvale S, Brinkmann S. Interviews: Learning the craft of qualitative research interviewing: sage 2009.
18. Krueger RA, Casey MA. Focus groups: A practical guide for applied research: Sage publications 2014.
19. ATLAS.ti Scientific Software Development GmbH. ATLAS.ti Web - The easiest way to uncover qualitative insights 2022 [Available from: https://atlasti.com/atlas-ti-web2022.
20. Burnard P. Writing a qualitative research report. *Nurs Educ Today* 2004;24(3):174-79. doi: 10.1016/j.nedt.2003.11.005
21. Burnard P, Gill P, Stewart K, et al. Analysing and presenting qualitative data. *Brit Dent J* 2008;204(8):429-32. doi: 10.1038/sj.bdj.2008.292
22. Hsiao B, Fraenkel L. Patient preferences for rheumatoid arthritis treatment. *Curr Opin Rheumatol* 2019;31(3):256-63. doi: 10.1097/bor.0000000000000591
23. Bywall KS, Kihlbom U, Hansson M, et al. Patient preferences on rheumatoid arthritis second-line treatment: a discrete choice experiment of Swedish patients. *Arthritis Res Ther* 2020;22(1):288. doi: 10.1186/s13075-020-02391-w [published Online First: 20201219]
24. Mathijssen EGE, Vriezekolk JE, Popa CD, et al. Shared decision making in routine clinical care of patients with rheumatoid arthritis: an assessment of audio-recorded consultations. *Ann Rheum Dis* 2020;79(2):170-75. doi: 10.1136/annrheumdis-2019-216137 [published Online First: 20191029]
25. Nagy G, Roodenrijs NMT, Welsing PMJ, et al. EULAR points to consider for the management of difficult-to-treat rheumatoid arthritis. *Ann Rheum Dis* 2022;81(1):20-33. doi: 10.1136/annrheumdis-2021-220973 [published Online First: 20210818]
26. Young A, Dixey J, Cox N, et al. How does functional disability in early rheumatoid arthritis (RA) affect patients and their lives? Results of 5 years of follow-up in 732 patients from the Early RA Study (ERAS). *J Rheumatology* 2000;39(6):603-11. doi: 10.1093/rheumatology/39.6.603
27. Hehir M, Carr M, Davis B, et al. Nursing support at the onset of rheumatoid arthritis: Time and space for emotions, practicalities and self-management. *Musculoskelet Care* 2008;6(2):124-34. doi: https://doi.org/10.1002/msc.115
28. Radford S, Carr M, Hehir M, et al. 'It's quite hard to grasp the enormity of it': Perceived needs of people upon diagnosis of rheumatoid arthritis. *Musculoskelet Care* 2008;6(3):155-67. doi: https://doi.org/10.1002/msc.132
29. Jones B, Bennett S, Larsson I, et al. Disseminating and assessing implementation of the EULAR recommendations for patient education in inflammatory arthritis: a mixed-methods study with patients' perspectives. *J RMD Open* 2022;8(1):e002256. doi: 10.1136/rmdopen-2022-002256
30. Knittle K, Maes S, de Gucht V. Psychological interventions for rheumatoid arthritis: Examining the role of self-regulation with a systematic review and meta-analysis of randomized controlled trials. *Arthritis Care Res* 2010;62(10):1460-72. doi: https://doi.org/10.1002/acr.20251
31. Astin JA, Beckner W, Soeken K, et al. Psychological interventions for rheumatoid arthritis: A meta-analysis of randomized controlled trials. *Arthritis & Rheumatism* 2002;47(3):291-302. doi: https://doi.org/10.1002/art.10416
32. Najm A, Lempp H, Gossec L, et al. Needs, Experiences, and Views of People With Rheumatic and Musculoskeletal Diseases on Self-Management Mobile Health Apps: Mixed Methods Study. *JMIR Mhealth Uhealth* 2020;8(4):e14351. doi: 10.2196/14351 [published Online First: 20200420]
33. Sirintrapun SJ, Lopez AM. Telemedicine in Cancer Care. 2018(38):540-45. doi: 10.1200/edbk_200141

For peer review only

34. Wosik J, Fudim M, Cameron B, et al. Telehealth transformation: COVID-19 and the rise of virtual care. *Journal of the American Medical Informatics Association* 2020;27(6):957-62. doi: 10.1093/jamia/ocaa067
35. Han L, Hazlewood GS, Barnabe C, et al. Systematic Review of Outcomes and Patient Experience With Virtual Care in Rheumatoid Arthritis. *Arthritis Care Res* 2022;74(9):1484-92. doi: https://doi.org/10.1002/acr.24586
36. Rodríguez Sánchez-Laulhé P, Luque-Romero LG, Barrero-García FJ, et al. An Exercise and Educational and Self-management Program Delivered With a Smartphone App (CareHand) in Adults With Rheumatoid Arthritis of the Hands: Randomized Controlled Trial. *JMIR Mhealth Uhealth* 2022;10(4):e35462. doi: 10.2196/35462 [published Online First: 20220407]

For peer review only

BMJ Open

Experiences of people with rheumatoid arthritis interacting with healthcare and their opinions on the use of a digital self-care application: a qualitative interview study

Journal:	*BMJ Open*
Manuscript ID	bmjopen-2023-072274.R1
Article Type:	Original research
Date Submitted by the Author:	15-Aug-2023
Complete List of Authors:	Viberg Johansson, Jennifer; Uppsala University, Department of Public Health and Caring Sciences<br>Blyckert, Hanna; Elsa Science<br>Schölin Bywall, , Karin; School of Health, Care and Social Welfare, Division of Health and Welfare Technology; Uppsala University Department of Public Health and Caring Sciences, Centre for Research Ethics & Bioethics
**Primary Subject Heading**:	Communication
Secondary Subject Heading:	Ethics, Health services research, Rheumatology
Keywords:	Rheumatology < INTERNAL MEDICINE, Self Care, Patient Participation

SCHOLARONE™
Manuscripts

I, the Submitting Author has the right to grant and does grant on behalf of all authors of the Work (as defined in the below author licence), an exclusive licence and/or a non-exclusive licence for contributions from authors who are: i) UK Crown employees; ii) where BMJ has agreed a CC-BY licence shall apply, and/or iii) in accordance with the terms applicable for US Federal Government officers or employees acting as part of their official duties; on a worldwide, perpetual, irrevocable, royalty-free basis to BMJ Publishing Group Ltd ("BMJ") its licensees and where the relevant Journal is co-owned by BMJ to the co-owners of the Journal, to publish the Work in this journal and any other BMJ products and to exploit all rights, as set out in our licence.

The Submitting Author accepts and understands that any supply made under these terms is made by BMJ to the Submitting Author unless you are acting as an employee on behalf of your employer or a postgraduate student of an affiliated institution which is paying any applicable article publishing charge ("APC") for Open Access articles. Where the Submitting Author wishes to make the Work available on an Open Access basis (and intends to pay the relevant APC), the terms of reuse of such Open Access shall be governed by a Creative Commons licence – details of these licences and which Creative Commons licence will apply to this Work are set out in our licence referred to above.

Other than as permitted in any relevant BMJ Author's Self Archiving Policies, I confirm this Work has not been accepted for publication elsewhere, is not being considered for publication elsewhere and does not duplicate material already published. I confirm all authors consent to publication of this Work and authorise the granting of this licence.

For peer review only

Journals: BMJ Open

Title: Experiences of people with rheumatoid arthritis interacting with healthcare and their opinions on the use of a digital self-care application: a qualitative interview study

Short title: Experiences of people with rheumatoid arthritis interacting with healthcare

Authors:
Jennifer Viberg Johansson, Hanna Blyckert, Karin Schölin Bywall

Jennifer Viberg Johansson (she/her), Associate Professor in Medical Ethics
Department of Public Health and Caring Sciences, Centre for Research Ethics & Bioethics, Uppsala University, Uppsala, Sweden
jennifer.viberg-johansson@crb.uu.se
+46 18 471 62 88

Hanna Blyckert (she/her), BSc
Elsa Science, Stockholm, Sweden
hanna@elsa.science

Karin Schölin Bywall (she/her), PhD
School of Health, Care and Social Welfare, Division of Health and Welfare Technology, Mälardalen University, Västerås, Sweden
Karin.scholin.bywall@mdu.se
&
Department of Public Health and Caring Sciences, Centre for Research Ethics & Bioethics, Uppsala University, Uppsala, Sweden

Corresponding author: Jennifer Viberg Johansson

Keywords: self-management, mobile application, patient perspectives, patient preferences, pedagogic program, rheumatoid arthritis

Word account: 9244

For peer review only

Funding

This project was supported by Vinnova, Innovationsfonden and The Research Council of Norway, under the frame of Nordforsk (Grant agreement no. 90825, Project NORA). The funding agreement ensured the authors' independence in designing the study, interpreting the data, and writing and publishing the report.

Competing interests

Karin Schölin Bywall and Jennifer Viberg Johansson have no conflicts of interest to declare. Hanna Blyckert is employed at Elsa Science as a Health educator and user researcher.

Data sharing statement

All data relevant to the study are included in the article.

Ethics approval statement

The study was approved by the Swedish Ethical Review Authority (Dnr: 2021-05431-01). Consent to participate was obtained from all respondents, both oral and written. This work was carried out in accordance with the Declaration of Helsinki and prioritised respondents' privacy and safety. When we requested consent, the respondents were informed that they could withdraw from the study at any time with no explanation and that the ending would not affect the current treatment. Their names were replaced with codes; all personal identifiers were removed so that the persons described, or narratives, were not identifiable.

Contribution Statement

JVJ, HS and KSB conceptualized the study. HB performed the interviews. JVJ and HB analysed the interviews and interpreted the data. JVJ was the major contributor into writing the manuscript. HS and KSB substantively revised the manuscript. All the authors read and approved the final manuscript.

Acknowledgment

Our greatest gratitude goes out to all the respondents who participated in the interviews. Thank you for your time and for sharing your experiences and opinions so generously.

For peer review only

Abstract

Objectives: Over the last decades, there has been great progress in the improvement of rheumatoid arthritis (RA) treatment. This relates to drug development and the development of guidelines for teamwork and patient self-care, and access to digital tools. This study aimed to explore the experiences of people with RA interacting with healthcare. Another aim was to explore opinions on how a self-care application, with an educational program, "The healthcare encounter", was improving patient-doctor communication.
Design: Semi-structured interviews were conducted and qualitative content analysis was performed.
Setting: The potential respondents, people with established, or under investigation of RA diagnosis, were asked to participate in the study via a digital self-care application.
Participants: Ten interviews were performed after the meeting with the rheumatologist or other healthcare personnel between September 2022 and October 2022. Phrases, sentences, or paragraphs with experiences from healthcare meetings and opinions about the digital program were identified and coded. Codes that reflected similar concepts were grouped; sub-categories were formulated, and categories were connected to their experiences and opinions.
Results: Among our respondents, we found three main experiences interacting with healthcare: the availability of healthcare, individual efforts to have a healthier life, and personal interaction with healthcare. Respondents described that the educational program can be a source of information, which confirms, supports, and creates a sense of control.
Conclusion: The respondents valued being seen and taking part in a dialog when they had prepared themselves (observed symptoms over time and prepared questions). The implementation of digital self-care applications might need to be incorporated into the healthcare setting so that both the patients and the healthcare personnel have a shared understanding. Collaboration is essential in this context.

Strengths and limitations of this study

- The study employed semi-structured interviews to delve into patients' perspectives on interacting with healthcare, allowing for a comprehensive exploration of their experiences.
- The study extensively investigated patients' opinions on the impact of a self-care application on patient-doctor communication, shedding light on the effectiveness and potential improvements in this crucial aspect of healthcare.
- This may not be transferable to other disease groups; it indicates how this particular group at this disease stage experiences healthcare.
- A limitation of the study was that only participants who used the app were invited to participate.

For peer review only - http://bmjopen.bmj.com/site/about/guidelines.xhtml

Introduction

Rheumatoid arthritis (RA) is an autoimmune, systemic, inflammatory, chronic condition causing joint pain and synovitis.(1) The disease affects around 0.5–1% of the population.(2, 3) Synovium happens in the joints due to the malfunction of the immune system. The disease typically affects one particular joint on both sides of the body, like both hands, knees, or ankles. RA also causes degenerative problems in other parts of the body, such as the eyes, heart, circulatory system, and/or lungs.(4) The disease usually develops over weeks and months. However, symptoms can come overnight as well. Many people experience unnatural fatigue, a general feeling of illness, as well as swelling and soreness in the hands and feet, since the disease often attacks small joints first. Morning stiffness for an hour or more is typical. (5)

In recent decades, significant advancements have been made in enhancing the treatment of rheumatoid arthritis (RA). These advancements encompass various areas, including the development of new drugs, the establishment of teamwork guidelines, fostering patient self-care, and improving accessibility to digital tools.(6) There is still no cure for the disease, but medications can reduce symptoms and help slow the progression of the disease development so that the person affected maintains good function in the joints. Several administration modes and medications can be combined to achieve the best effect on the symptoms. The treatment needs to be adjusted throughout life because of new symptoms, lack of effectiveness, side effects, or other components. Besides effective medication, other components of the RA treatment need to be considered such as education about self-care and self-management to increase adherence to medication. Communication skills are also important for rheumatologists and other healthcare personnel to promote shared decision-making between the patient and the rheumatologist and enhance patient empowerment.(7)

Apart from the physical changes resulting from RA, people also face many psychological challenges like distress and helplessness.(8) They need to cope with a general feeling of being unwell, which can result in decreased ability to participate in daily life, social, and recreational activities. Psychological well-being (including e.g., self-esteem, self-perception, self-worth, body image, relationship with their partner, and limitations in sexual activity) has been shown to correlate negatively with the level of joint tenderness in people with RA.(9)

Besides finding the right medication use and hindering the development of disabilities in the joints, guidelines recommend providing education and tools for handling psychological aspects of the disease.(4) There are recommendations to help patients identify factors and means to manage symptoms like pain, fatigue, depression, anxiety, and sleep problems.(7) Earlier research has emphasised the need for education in self-management skills and counselling to enhance empowerment and improve the emotional dimension of care. Findings support the need for team-based rehabilitation interventions to enhance empowerment in patients with RA.(10, 11) Moreover, results from a review of RA patients' self-management support needs show that people with RA have informational, emotional, social, and practical needs. Informational needs can be about how to deal with pain, exercise, and medication. Emotional support can be from their relatives or healthcare professional.(12)

Using mobile technology as a complement in healthcare has increased during the last decade and is now a natural part of healthcare-related services in general,(13, 14) as well as for people with RA who require daily self-management of the disease.(6) The shift towards using digital tools has changed healthcare in many ways. RA patients can now inform themselves

For peer review only - http://bmjopen.bmj.com/site/about/guidelines.xhtml

using trustworthy sources, log their status and self-manage the progression of the disease without having to be at the hospital. The digital tools come with a promising opportunity for self-management and can be helpful in preparing for interactions with health care professional/provider. Moreover, adding digital tools has been shown to be more effective for treating RA and is therefore also cost-effective.(15) However, comparing the traditional care with the more collaborative care, a more shared expertise is needed between the lived experience of RA and healthcare knowledge. Both collaborative care and self-management education shift focus onto people's own responsibility for their health. Yet, great responsibility remains with the healthcare personnel, who have an active role in communicating, activating, and assisting patients in the self-management of their condition.(16)
The medical evidence, international and national guidelines and legal prerequisites, and digital technologies have been rolled out to enable good care of RA patients. The question is whether the healthcare in Sweden is ready for a more active patient and whether such interactions between people with RA and the rheumatologist work well in today's healthcare. There is a need to explore how people with RA experience interaction with health care professional/provider and the use of digital tools to improve self-care and communication.

This study aimed to explore the experiences of people with RA, or under investigation for RA, interacting with healthcare. Another aim was to explore opinions on how helpful a digital self-care application was in improving patient-doctor communication.

For peer review only - http://bmjopen.bmj.com/site/about/guidelines.xhtml

Methods

Design

The study was a qualitative semi-structured interview study.

Respondents and the setting

The potential respondents, people with established, or under investigation, RA diagnosis (Swedish guidelines for diagnosis) and being affiliated with a rheumatology clinic, were asked to participate in the study via a digital self-care application called Elsa (https://www.elsa.science/en/), see supplementary file named Example of App Content.pdf. The access of the app were from both Google Play Store for Android and Appstore for iOS. They found it out via the clinic (recommendation by staff or brochures at the health centre), via digital platforms and social media, or by their own search for self-care. Respondents were eligible for the survey if they had an RA diagnosis, were aged 18-80 years, and understood and expressed themselves in Swedish. The structure of the study was to first participate in a survey, then perform a specific program in the Elsa app called "The healthcare encounter" and, in conclusion, do a final survey. In the final survey, they had the chance to sign up for a follow-up interview. It was voluntary to sign up.

The specific program aims to provide basic knowledge to individuals living with a rheumatic disease that can inspire them to make sustainable lifestyle changes and improve their well-being. "The healthcare encounter" program focuses on providing basic knowledge about the treatment, medical options, what to expect from meeting healthcare, and how the individuals can prepare themselves before the meeting. This knowledge program took about 20 minutes to undergo. Each person could decide for them-self whether they would like to do it all at once or split it over time. The daily log can be spent around 2-3 minutes per day. See Box 1 for the content of the knowledge program.

Box 1.

**The content of the educational program**
- Introduction, background, and basic knowledge<br>- The goal of the encounter (treatment goals)<br>- The treatment in general (different medication options)<br>- Long-term collaboration (the healthcare system's role and your role as a patient)<br>- How to prepare (before the encounter)?<br>- How to act during the encounter?<br>- How to follow up after the encounter?<br>- Checklist

Data collection

Eighteen out of 43 persons declared their interest in participating in this interview study. The respondents were then asked via e-mail to schedule an interview. The study included participants who had appointments scheduled within the near future or within the past month. Ten interviews were performed after the meeting with the rheumatologist or other healthcare personnel between September 2022 and October 2022 by the second author (HB).
The interviews lasted 44 to 63 minutes and were conducted in Swedish via Google Meets or telephone (n=1). We began each interview by asking about their experience of having RA, their treatment, and health in general. Thereafter, we asked them to describe their experience

For peer review only - http://bmjopen.bmj.com/site/about/guidelines.xhtml

before, during, and after the healthcare meeting. They were also asked to describe the use of the digital program *The healthcare encounter*, as to whether it was useful to them or if it was missing any information or function. A semi-structured interview guide with open-ended questions(17, 18) was developed with all authors; see Table 1. The interview guide was subjected to testing among colleagues and an individual with RA to assess question comprehension. The sequence of questions was reorganized to align with the temporal aspects of preparations and experiences before, during, and after the medical appointment and the use of the app.

Table 1. Interview guide used for the semi-structured interviews

Description of the project, the goal, and our background knowledge and interest.<br>Can you please tell me about yourself and your life at the moment (warm-up question)
Before your appointment with healthcare provider– how did you feel before your visit?<br>Probing questions: Did you prepare in any way? If so, how? Was there anything specific you planned to bring up at the meeting? Did you get any help from the program in your preparation? If so, what? Was there any difference in how you prepared now compared to how you usually prepare yourself?
During the appointment with healthcare– how was the visit?<br>Probing questions: What did you talk about (feeling in general, treatment, side effects, etc.)? What felt good/less good during the meeting? Did you have the opportunity to bring up what you wanted to talk about (treatment options, any side effects, other complaints, what is important to you)? Compared to previous visits, how did you experience this?
After the appointment with healthcare– do you remember how you felt after the visit?<br>Probing questions: Was the meeting as you wanted it to be? Would you like to change anything if you could in retrospect? In retrospect, is there anything that feels unclear after the meeting (missing info, unsure of how to do something, proceeding with self-care)? How have you handled the uncertainty?
About the program– what do you think about the program?<br>Probing questions: Was there something that you particularly liked? Something you liked less or even disliked? Is there something you missed or would like more of?

Analysis

The recorded interviews were transcribed verbatim by a professional transcription company. The transcripts were listened to in their entirety to verify the transcriptions. After all the transcripts were read again, meaning units (phrases, sentences, or paragraphs), with experience about healthcare meetings and opinions about the digital program, were identified for further scrutiny. The material contained 344 meaning units in total. Atlas.ti Web(19) and Microsoft Excel (2016) were used to assist in the data management and analysis process. In the next stage of the process, we continued with comparisons of the meaning units, examining their similarities and differences from the perspective of experiences and options of healthcare and the digital program. Open coding of each meaning unit was added, which summed up what was being said in the text. Two of the interviews were coded simultaneously by both authors (JVJ and HB) who jointly discussed what meaning units to identify, interpretations, and formulations of codes. The rest of the interviews were coded by JVJ. Codes that reflected a similar concept were grouped; sub-categories were formulated, and categories were identified(20, 21) by JVJ and thereafter discussed thoroughly with HB and KSB; see Table 2. Thematic saturation was reached about the aim of the data collection. The authors JVJ, HS, and KSB bring several years of experience and in conducting interviews and analysing qualitative data. Field notes were taken both during the interviews and throughout the analysis process.

For peer review only

Table 2. Example of the analytical process of the experiences when interacting with healthcare.

Meaning unit	Initial coding	Sub-category	Category
But like [trying to get in touch], you just feel, what the hell, I have no power.	Does not reach care	Accessibility and confirmation of care	Availability of healthcare
I have expressed several times to them on the phone, I feel alone. I feel very alone. I think it is hard actually. But then, it's not like I'm lying down and crying about it, but I feel left out.	Feeling alone and left out	To be taken care of	Availability of healthcare
He is responsive, he listens, understanding […] it is a pleasure to go and see him. I never feel any stress or pressure when I talk to him. He is very responsive and listens... he always takes what I say seriously.	He is responsive, listens, and explains; he takes what I say seriously.	To be seen and met with interest	Personal interaction

Patient and public involvement

None.

Results

In total, 10 interviews (seven females, three males) were conducted with persons with RA, or under investigation (n=1 under investigation with symptoms two years back in time and later after the interview that person received a confirmed diagnosis of RA). Disease duration ranged from one to three years, with one exception, where the person received a diagnosis 12 years ago. The respondents were aged 45–76 years (mean age 56.7) and from different demographic locations in Sweden. All participants in the study were taking medication, and they were recruited from across the entire country. All participants were accustomed to using smartphones and mobile applications in their daily lives. All respondents described their experience of being diagnosed with RA as a challenge. Those who were still unsure of what their symptoms indicated expressed frustration about not knowing or understanding the body's signals. They had tried various healthcare services that might be helpful, and also medications that might work. Having problems linked to other diseases was also described as a challenge. Consequently, they described having to choose which health issue to alleviate first. Accepting the disease was experienced as both a challenge and a necessity. What was also described as a challenge, and a necessity, was finding a balance in life between activity and recovery, work and leisure, and physical activity and rest.

The next section describes the respondents' experiences of interacting with healthcare. The qualitative content analysis revealed three main categories. Thereafter, nine sub-categories were used to classify the discussions. The categories were: 1) Availability of healthcare, 2) Individual effort, and 3) Personal interaction. An overview of the categories and subcategories is presented in Table 3. Below, these categories and sub-categories will be described and illustrated by quotes.

For peer review only - http://bmjopen.bmj.com/site/about/guidelines.xhtml

Table 3. The categories and sub-categories of respondents' experiences interacting with healthcare.

Category	Sub-category
Availability of healthcare	To feel prioritised
	Accessibility and confirmation of care
	To be taken care of
Individual effort	Taking care of myself
	Opportunity to equip me
Personal interaction	To be seen and met with interest
	Gain self-efficacy
	Have a dialogue
	Met with competence

For peer review only

Availability of healthcare

The category *Availability of healthcare* was expressed in three different ways: *To feel prioritised*, *Accessibility and confirmation of care*, and *To be taking cared of*

To feel prioritised

This sub-category is about the experience of feeling that there is enough time for patients and healthcare personnel to discuss symptoms, treatment options, and whether resources are invested in helping this group. About half of the respondents described they felt prioritised and had a great team around them and great support from different healthcare personnel. The rest of the respondents expressed a feeling of not being prioritised in situations when they met healthcare providers. They stated that the care meeting was too brief so that one simply did not have time to discuss things related to the disease, besides medication; moreover, they also could not ask follow-up questions. The time constraint was perceived as preventing a personal meeting. Some expressed a feeling of not being treated like a human being; it was more like an assembly line.

> *Well, I would have liked his [the rheumatologist] clinical part first[...] go through the joints and any blood tests and explains what the result means, so that you might learn something yourself. So you don't have to keep asking more times. And I wish him to ask, 'How does everyday life work for you?' I still have not been asked that question since 2019. (Respondent 3)*

Some respondents suggested the benefit of having more frequent meetings early on in the process when more issues arise and there have concerns. The possibility of having more meetings, if needed, gives the impression that you are important. Being met by healthcare personnel who do not know about rheumatic disease can make you feel insecure, resulting in a feeling that this patient group is not prioritised.

> *..then I felt some insecurity. If it were someone who had the actual expertise, then they may have asked other follow-up questions. The person was very nice, but it felt a little unsafe. It felt like, who is taking care of me? It's not her fault, of course, but that's how I felt...(Respondent 7)*

For peer review only - http://bmjopen.bmj.com/site/about/guidelines.xhtml

Accessibility and confirmation of care

Having healthcare that is accessible is highly appreciated and valuable for the respondents. The sub-category focuses on access, contact, and knowing where to seek assistance. Some expressed that it was easy to make contact via phone or messages via the digital platform used in Sweden (named 1177). When problems with symptoms or side effects occur, fast response, action, and reply are appreciated, giving a calming feeling. Having regular meetings scheduled also led to feeling safe.

> *I think it [meeting the rheumatologist] feels safe. I think it feels great. So, I'm happy to continue with that. (Respondent 5)*

However, not all respondents experienced this. Most felt it was difficult to reach healthcare via phone or messages. One described it as a whispering game; you call and tell a healthcare personnel the problem and that message is forwarded to the rheumatologist, who then sends a message back via a third person. These difficulties in reaching healthcare are perceived as unsafe care, resulting in the feeling of being exposed to danger.

> *... and I know that care is generally heavily burdened, so without talking badly about an individual. But like, you just feel, what the hell, I have no power. (Respondent 7)*

The fact that the care provides confirmation is also highly appreciated and valuable to the respondents. Some described that the rheumatology clinic does a fantastic job and that you, as a patient, get quick answers if you have concerns or experience practical obstacles with medications. They also stated they receive a good follow-up of blood test results. However, for other respondents, it can take a long time to get confirmation of test results. Sometimes it is not until the next appointment or if they have indicated they are not doing well.
The respondents who experienced collaboration regarding the treatment of RA expressed that the various professions had good accessibility and followed up on their work, and it was clear how to reach out to them when a problem occurred.

> *I have to say that I am very impressed with the rheumatologist at the hospital [...] and I do that [send a message] and it can take within an hour then I've got a response that either the nurse has answered, or she says that "I've passed it on to your doctor" and then in the afternoon, I get an answer. So, this is how it has been for these four years, fantastic. (Respondent 5)*

To be taken care of

All respondents appreciated having healthcare personnel who conveyed a feeling that one is being taken care of. Some respondents expressed great gratitude and were impressed by the teamwork they had experienced. They felt getting the right support gave a feeling of being taken care of, trustworthiness in the care, and the feeling of having support if difficult times were to come. A noble example, told by one of the respondents, was when the healthcare profession helped to refer her onward, instead of letting the patient seek help again on her own. The respondents experienced great teamwork and expressed pride in their care unit, stating that the staff had a nice and welcoming attitude, and the feeling of being looked after was strong.

For peer review only - http://bmjopen.bmj.com/site/about/guidelines.xhtml

…then he [the rheumatologist] had been talking to the gastroenterologist and discussed with them and put together an action plan with them. (Respondent 6)

Some respondents had the opposite experience: frustration at being sent around or not getting good follow-ups on their symptoms, blood tests, or medication. They expressed that it caused concern and created incredible insecurity as to whether someone had control over the situation. The respondents asked themselves if they did not hear from the healthcare system, what does it mean: that they do not care, that they have forgotten about me, or that the test results are without remark? It said that not hearing from healthcare resulted in a feeling that no one watches over you.

Some respondents felt that you needed to time it, so you were sick enough at the meeting to receive the right help. Being well at the time of the appointment resulted in not getting the right help. Because symptoms come and go, some respondents found it difficult to find an appointment time when they were bothered; consequently, they did not feel they were being taken care of.

And maybe also some form of follow-up. I mean, if he doesn't find an inflammation now, I might have it in a month or a week. That they sort of had some kind of... yes, but "if you get an inflammation, get in touch and come here and then show us, and then we'll get... and then we'll take new measures". Instead of just ending the meeting and then I am sent back to primary care. (Respondent 8)

Knowing where to turn and receiving help was perceived as important by the respondents and gave a feeling being taking care of. Some described they had access to direct numbers if they had a flare-up. Some expressed they had a good routine for sending a message to the rheumatologist, who replied to them quickly. Others expressed difficulties in that they were sent around in healthcare: Primary care to different specialists and then back to primary care. For respondents who had many different ailments, where it was difficult to know what was in the illness, they talked about the difficulties in finding the right help. They described a long journey to finally receive help for their symptoms and a diagnosis. They claimed it was difficult to understand the course of healthcare; moreover, they were frustrated that different healthcare units did not communicate with each other. Some respondents talked about how they had to be the ones who coordinated the care. This was perceived as tiring and difficult. Finding the right care can be an obstacle and take a lot of endurance. When it was not clear what treatment works and what symptoms to prioritise, the respondents thought it took a lot of effort and energy. Respondents who had many symptoms and also experienced other diseases at the same time expressed great difficulty in finding the right care in complex situations. One respondent considered changing to a bigger hospital to receive better care, having to consider a longer journey.

[…] Why should I change [health care unit] then? …as I understand it, it is a bigger hospital. After all, there is a larger clinic and more doctors who are specialised. Often, when I call my specialist's office, it's... well, it's the diabetes nurses I get to talk to, it's like... I do not know what it is like in there [at the bigger hospital], but it sounded like it could be different. So I

For peer review only - http://bmjopen.bmj.com/site/about/guidelines.xhtml

said, of course, I want to change because I want to be where I get the best help; of course...(Respondent 7)

Some felt they have been sent around to different units to have their concerns heard. It can also be difficult for a patient to know where to go with the problem that is occurring: is it the nurse, the physiotherapist, the occupational therapist, the curator, or the rheumatologist?

[…] it is not crystal clear, where to go in healthcare. And I'm a migraine person. A lot of small problems. But I'm a very stubborn person, and I don't give up. I fight.

[…] You are sent around a lot between different healthcare units, depending on what problems arise. And there is no connecting link. One part of healthcare does not know what the other part is doing. (Respondent 6)

Individual effort

The category of *Individual effort* was expressed in two different ways: *Taking care of myself* and having the *opportunity to equip me*.

Taking care of myself

All respondents expressed they have tried to adapt to life in different ways and undertake different attempts at self-care: they have adjusted their diet, tried different forms of exercise and training, and tried to find a balance between rest and activity. The respondents described how they prepared themselves in different ways before the visit for the conversation with the rheumatologist. In addition to taking notes on how they feel – either with pen and paper or via digital tools – they sought information in different ways to equip themselves with knowledge. Some stated that equipping themselves with new knowledge was for their own sake. Others stated it was also to facilitate healthcare, so they can be involved in the 'detective work' of finding effective treatment. Moreover, they try to understand and interpret thein test results on the health and medical care's digital platform (name 1177) before the meeting. Writing down questions, symptoms and how they have felt over a longer period were common actions. This was, for some, useful as a tool to reflect on their health, understand the reasons for the symptoms, and how they come and go, but also to remember the ups and downs of their symptoms.

I had a long list I had written before the phone meeting, where I tried to think "How long have I been in pain", "Where do I have pain", "how do I react" and "what medicines have I taken" and so on. So that I don't forget anything when I talk to the physician. (Respondent 6)

Some expressed frustration that they must know a lot themselves to get help, that you have to stand up for yourself to get good help, and that it feels like one has to be healthy to be able to be sick.

I don't think it's okay, I told her […] all the side effects. Then it was like "Well, okay, let's try something else". I have had to argue a lot to change my medication… (Respondent 5)

For peer review only - http://bmjopen.bmj.com/site/about/guidelines.xhtml

A few participants expressed a positive feeling about taking part in research in the field of RA. They described it gave a nice feeling of being able to give back to healthcare for all the good things they have received.

Opportunity to equip me

There was great variation in the extent to which our respondents had the opportunity to be educated regarding their disease. Some had four days of education, some had one day or even half a day, and some did not receive any education from their rheumatology clinic. To receive education about the disease, medication, exercise, lifestyle habits, and how to find a balance in life was much appreciated by the respondents who experienced that. They felt it made them feel safe and satisfied even though life has changed with the diagnosis. Education was perceived to empower them and made it possible to take care of themselves.

> *... the introductory education... I mean, four full days. And so, they have it regularly [...] with everyone who is newly diagnosed, where they go through [everything]. And they also went through which medicines are available, and said "And we start here" [...] They were very clear that we start here and then you have a period where you test it and if it doesn't work, then we move on to something else. So they informed me about the whole process. (Respondent 6)*

Those who did not receive education from the rheumatology clinic expressed a desire for it and hoped and even expected healthcare to provide it. Some described with surprise and disappointment that they had not received it. Understanding one's illness is deemed important; one wants to understand and take power over one's life. Specific aspects for which they wanted more knowledge were medical treatment, side effects, diet suggestions, diagnosis, and what to expect from life.

> *...training and such, they talk about that and what is good. That you shouldn't smoke and like... yes, and so on. But diet, in particular, seems to be taboo. (Respondent 8)*

Personal interaction

Finally, our analysis revealed experiences related to personal interaction, and how the healthcare professionals' attitudes made respondents feel. This is the most comprehensive category regarding the participants' narratives. This category was expressed in four different ways: *To be seen* and *met with interest*, *Have a dialogue*, *Gain self-efficacy*, and *Met with competence*.

To be seen and met with interest

There was a strong emphasis on the appreciation of being seen, namely that professionals would meet the patient with empathy. The respondents focused on the personal interaction with the physician and whether one feels individually attended to. Respondents wanted to feel as though they were believed, not perceived as whiny, and to gain some hope in difficult situations. They wanted to be seen as a whole individual and talked to as an equal, and that personnel did not look down on them. When the doctor takes the time and listens, the feeling of being seen as a person increases.

For peer review only - http://bmjopen.bmj.com/site/about/guidelines.xhtml

Very professional, very calm, and nice and she gave me hope. (Respondent 5)

Some stated they felt stupid when interacting with the rheumatologist. They emphasised the importance of including the patient's perspective, or at least having a nice response. Not being listened to, resulted in a despondent feeling as if "it doesn't matter what I do". It also led to a cold, not human, robot-like feeling in the relationship. Respondents stated that if one has prepared and written down how they have felt lately, then it is important that this information is not overlooked. Focusing only on medicine and side effects, and not any other aspects of the disease, was not viewed positively. In such cases, respondents felt they were not seen.

... they don't know me. I've met him once, and yet, they somehow assume that… you need to bite the bullet. It's a bit like that. And the first time ever when I was at the health center, when my fingers hurt... and then I had red knuckles. They were really inflamed. Then the medical centre doctor told me that I have to learn to live with that, you just have to bite the bullet. (Respondent 8)

The feeling that the rheumatologist is interested in the patient's story and takes time to answer was expressed as a valuable thing. The professional approach was important to patients, where the attitudes and responses of healthcare personnel can help patients feel respected. Being on time, listening, and meeting the patient's expressed needs, gave a feeling of respect. If the healthcare does not call back as they promised or does not write down the patient's medical records correctly, or not at all, it is a disappointment. This was viewed as the health care professional/provider not being interested in them, that they are just one on a product line. Nevertheless, the fact that the rheumatologist is well-read and prioritises the right things to discuss at the meeting was perceived very positively, making the patient feel as though he or she is seen with interest. Respondents expressed a positive attitude when they were seen as a resource; the doctor is the medical expert, while the patient is the expert on themselves. They felt that for the encounter to be as good as it could be, they both needed to share their expert knowledge.

…since she is so calm and takes her time and listens to me, I don't have to stress through them [the questions] or skip any. I may have twenty questions, of which five are very important, and fifteen are... if we have time, I will ask them. Then I can get answers to something that I have wondered for years. (Respondent 4)

Some respondents with bad experiences with healthcare described they felt that for the rheumatologist, they are just a body to be examined. They felt they are not allowed to talk about where they have pain and for which symptoms they would like help to alleviate. One participant felt that her own illness story was uninteresting to the rheumatologist. Another respondent expressed she sometimes feels that she needs to lie about her current pain during a healthcare visit to get help, even if the flare-up of pain this time has just passed. This is because she is worried that her complaints will not be taken seriously just because she is feeling a little better at the moment. The meeting with the doctor is a cross-section of the person's life and that is frustrating to deal with. There is a wish to understand on what grounds the physician makes his or her medical decision. The respondents expressed a wish

For peer review only - http://bmjopen.bmj.com/site/about/guidelines.xhtml

that the lived experience of the symptoms and the side effects can be used in the treatment discussion.

> *… the physician says that the physician's task is to fix the medicine and I take care of me, what is best for me. But then I have to tell the physician how I feel when I do certain things. But no one ever cares about that, it's just, what does the medicine say, only the blood measurements count, how does it feel in the finger joints, period. (Respondent 6)*

Have a dialogue

The respondents appreciated having a good dialog with the rheumatologist; being able to reason with the rheumatologist about medical treatments, and side effects and finding a balance between things in life were perceived as valuable. Moreover, the respondents appreciated the rheumatologist taking advantage of the preparation done by the patient before the meeting. Being able to reason about the amount of medicine was also appreciated.

> *I'm a bit fussy, I don't want to take tablets that much, so I stopped after a while, not with Benepali because I understand that I need it. But these painkillers […] but now for a period, it hasn't been good and then my doctor said yesterday "One more tablet isn't dangerous and it's not much" […]. So then, I thought, okay, now I have to listen to her. It might have something to do with the fact that I may not have fully accepted this, I thought last night, because I'm so fussy and don't want to take the medicine. (Respondent 5)*

Those who did not have the opportunity to have a good dialog, they expressed sadness or frustration about what was missing for them. They all described they had heard that other patients had received better care. When a good dialog was not present, the respondents felt they did not trust the rheumatologist, and either wanted to change the contact person or go their way when it comes to medication choices or lifestyle changes. For some, it was not clear whether it was ok to be critical or even change their rheumatologist if the communication does not work out well; it might result in bad consequences.

> *So, actually, I would like to change my physician. Because there is another doctor there too who helped me at some point when I had pain in my joints in the palm. He was very nice, accommodating and calm, and such. So actually, I want him. But then you don't know if you dare to change the physician like that, because then [...] what if I get the evil eye […]. So, I feel, it's better I go there to those meetings with him, and he does what he has to do, then I google and learn by myself. (Respondent 3)*

There was a feeling of resignation among those who prepared but were not listened to. Even though they prepared well (what to ask and how they have felt during the past month), the rheumatologist did not take notice of that: they shared that the doctor did not even look them in the eyes; wasn't interested in the patient's notes or questions; and answered using difficult language. Instead, the rheumatologist went on with a clinical test and made some comments about the medicine. These respondents felt they were being ignored and could instead be a valuable resource.

For peer review only - http://bmjopen.bmj.com/site/about/guidelines.xhtml

[…] even though I had kept a log for a very long time, there was nothing that he [the rheumatologist] cared about when I was there. So, he looked there and then, the day I was there. "Are you in pain here, yes. Are you not in pain here, no"? And then it was fine with that. (Respondent 8)

Gain self-efficacy

Something that was expressed by all respondents was the need for encouragement and hope for the future. Healthcare was perceived as an important component in the respondents' attempt to find a functional life. It is important to be able to express yourself and be taken seriously; then, there is a feeling of being competent in dealing with the new situation. For some, meeting healthcare can often create a feeling that one has become old and dying. Getting help to set new goals and sub-goals was perceived as helpful; it helps to believe in the future.

… I have thought about it quite a lot and that helps me, that I need to have a goal. Not a dream, a goal. Everyone has dreams, but then you can pick certain dreams and say that is a dream, and this is my new goal. The goal for me is to get back on the police motorcycle. And being able to function and not have to think about how I'm paddling a kayak with my wrist or how I'm holding the weight or the dumbbell, or whatever it is. But I should just be able to be, as I was before. (Respondent 3)

Met with competence

If the rheumatologist is knowledgeable and competent, it infuses confidence in the patient. Some respondents are impressed by the rheumatologist's skills in balancing the trade-off between deploying different medications at different times. The respondents felt they get all the answers they need to remain calm and reassured, and following up on any side effects or symptoms was appreciated.

…questions about the medicine to the rheumatologist... she is good at explaining. (Respondent 4)

Two respondents felt they received better care if they were prepared and knew about things themselves. Some respondents, however, were disappointed as they did not receive answers to their questions and felt the rheumatologist was only guessing when responding to them, resulting in them feeling resigned.

No, but he seems not to care; it feels so stiff when I talk to him. I had to remind him […]. Well, he's not well-read and that's what annoys me so much when he calls. I know in my heart that he is not well-read. (Respondent 10)

For peer review only - http://bmjopen.bmj.com/site/about/guidelines.xhtml

Opinions about a digital self-care application

A further aim of this study was to explore opinions on how helpful a digital self-care application with the educational program was to improve respondents' ability to express themselves in patient-doctor communication. They expressed that a digital self-care application can be a *Source of information*, and help to *Confirm*, *Support*, and create a *Sense of control*.

Source of information

Many respondents appreciated the application as a trustworthy source of information with a good spirit.

> *I think it is great [...] easy to understand and at a good level. And it's good to be able to go back and remind myself. (Respondent 9)*

> *So, I google... being critical of sources, it's not that easy as a consumer. This [the application] feels very serious and here they have talked to people who [are knowledgeable and experienced] ... that's the image I have, anyway. (Respondent 7)*

They valued being able to learn more about what to expect with the disease, to learn about the medication, different healthcare personnel's responsibilities, how to inform relatives, and what activities to engage in to feel better. Learning from others' experiences was also appreciated. One expressed that the app could be useful even if one was not yet diagnosed.

However, two respondents expressed that the information was not a surprise and that it confirmed what they already knew. One respondent believed the content did not always have to be so gentle and nice all the time – life is hard.

Confirm

The respondents claimed that using a digital self-care application creates the feeling of being seen, that someone is carrying and confirming one's experiences. By reading what others have been through, your own experience is confirmed, and you become calm.

> *Other people's stories in the app, […] I just… Yeah, but damn. That's me! (Respondent 4)*

They expressed a positive attitude when someone answered their question, via e-mail or the chat function, that someone was replying to their questions and concerns regarding the progression of the disease. It was also mentioned that it is good to digest information and to ask questions at one's own pace, not being rushed or stressed that it is now or never you get the chance to express your concerns. However, one respondent stated that the application's content was not reflecting that person's reality; the reality of how well healthcare works is not as good as described in the application; the app description of healthcare was "too good to be true".

> *[...] the description [in the application] where the doctor looks at the clinical aspects and then I help explain how I feel and how I experience everyday life and what works for me and what doesn't work. Yes, but it was*

For peer review only - http://bmjopen.bmj.com/site/about/guidelines.xhtml

something that I got hooked on and immediately thought yes, but I haven't had the chance to experience that yet. Such a doctor's visit. (Respondent 3)

Support

The digital self-care application was perceived as a good tool to help prepare before the meeting with healthcare. It also strengthened one's self-esteem before meeting with healthcare.

So yeah, I thought it [the content in the application] was... it helped me believe in myself. (Respondent 8)

After the respondents have gone through the program, they are given a list of good things to prepare for the meeting in the healthcare setting. This list was found to be very helpful to read and think about before the meeting. For the patient, there can be so many thoughts and feelings (e.g. fear, frustration, joy) so it can be difficult to focus on the most important parts. Therefore, they viewed the checklist as a good tool to not forget what is important and help to not lose focus. It also confirmed that it is good to be prepared. To be able to look back on the personal log to remind themselves what had happened over the last period was also appreciated: that was perceived as a good memory support tool. One respondent commented it would be even better if the digital self-care application could invite or push for healthy lifestyle choices. Another suggestion for the application was to include personal goals and help to set interim goals. The function of logging symptoms was also viewed as a good support or basis for reasoning with the rheumatologist. Furthermore, some wished that healthcare had access to their logging data to be able to have better conversations over the previous period and discuss treatment options and outcomes. However, some thought that being constantly reminded of one's illness via the log or reading about all the negative things about the disease can weigh one down.

...there is a risk that going on and on like that [with reading and log symptoms every day]... that you dig into your illnesses, and I don't think that feels very good. (Respondent 2)

There was also a positive attitude towards including information and educational material to and by the relatives. It can be difficult for someone affected by symptoms to share that without sounding negative and as if they are complaining. It was stated that information directed to the relatives could ease the burden and facilitate good relations in the family.

Create a sense of control

The respondents thought the app was fun, helpful, and a way of taking care of oneself when filling in the daily log. For some, it gave them time to reflect on how they feel and how the symptoms changed over time. Some thought it would be helpful to log long-term pain, diet, physical activity, general mood, and sleeping pattern. They also expressed a need to be more specific in describing the exact location of the pain (e.g. where in the hand). Moreover, they suggested making a note of the possible reason for the pain by typing the activity performed before the pain occurred. On the other hand, some respondents said they forgot or felt they did not want to log their symptoms when everything was fine.

I usually go in and use it [logging symptoms], although it's become a bit more sporadic now the last... well, last month and so. Because I have felt

For peer review only - http://bmjopen.bmj.com/site/about/guidelines.xhtml

better and then when I feel better, I kind of forget to fill it in somehow. (Respondent 9)

Some respondents pointed out that it was a challenge to log in information when you have symptoms that are not included in the app, for example, neck pain or migraine. Another challenge was distinguishing between what is a symptom due to the illness, ordinary fatigue, or pain from another cause. Respondents thought it was difficult to be objective. Many of them wanted to enter even more symptoms on the daily log, related to multiple illnesses to understand why the pain arises, to unravel the mystery to find good coping strategies and minimize their symptoms; like performing detective work.

For peer review only

For peer review only - http://bmjopen.bmj.com/site/about/guidelines.xhtml

Discussion

This study aimed to explore the experiences of people with RA, interacting with healthcare. People with RA are a well-studied group when it comes to the exploration of clinical outcomes of disease-modifying antirheumatic drugs (DMARDs) and patients' views on those clinical outcomes.(22, 23) This study brings new aspects to this perspective by asking people with RA about their experience of interacting with healthcare. It is well-known that making shared treatment decisions plays an important role for persons with RA to improve clinical outcomes and their adherence to treatment in the way the DMARD is prescribed.(24)

Our study highlighted the importance of patient-centered care, specially focusing on meeting patients' psychological needs and over all well-being alongside their physical health. Our respondents valued time and dialog with the rheumatologist. They appreciated and felt it was useful and necessary that their own lived experience of the disease was considered when evaluating symptom relief and discussing further treatment. According to the EULAR recommendations, discussions to introduce the patient perspective could benefit both the patient and healthcare by optimising self-management and treatment adherence.(25) Besides the physical changes that are inevitable with RA as well as the medical challenges, people with RA face many psychological challenges like distress and helplessness.(8) Our respondents expressed the need to accept the new situation. They also requested help from healthcare to do that. We interpreted that those respondents who had a good experience of teamwork, with several healthcare personnel included in the treatment, had a calmer approach to themselves. We also interpret that if the patients know what to expect from the meeting, when the next meeting is, and who to turn to when symptoms increase, it gives them calmness, which is beneficial for the disease activity overall as well. We recommend, to enhance disease management and overall well-being that healthcare providers ensure that patients with RA, and including those undergoing investigations possess a comprehensive understanding of the healthcare team's plan and expertise, achieved through clear communication and education regarding the treatment plan, expectations, and available support.

Given that psychological well-being has been shown to correlate negatively with the level of joint tenderness in people with RA,(9) a more holistic approach is needed. The respondents in this study expressed both positive and negative experiences about including well-being issues in the meeting with the rheumatologist. Common reactions to the diagnosis of RA include feeling overwhelmed and that life has taken a new, unexpected, and unwanted turn. Finding out that you have a lifelong chronic disease can make you sad, and create anxiety and fear for the future,(26) thus perhaps requiring appropriate professional support. There is a risk that once diagnosed, the immediate focus becomes finding the right drug therapy to reduce inflammation and pain. Finding the right drug is challenging and can take months. After testing a drug, the next follow-up appointment primarily focuses on the efficacy and monitoring of drug therapy.(7) Therefore, it is important to meet those psychological needs as well, and not only look at the joints, inflammation, test results, and X-rays. Some of our respondents expressed frustration at not being seen as an individual; just a body that needs to be examined. This is not in line with the recommendations(25) and should not occur.

Building on the need for self-management skills, it is crucial to address the informational, emotional, social, and practical needs of individuals with RA. Previous studies and guidelines have highlighted the importance of education and counseling to empower

For peer review only - http://bmjopen.bmj.com/site/about/guidelines.xhtml

patients and improve the emotional dimension of care and guidance on how to live with RA.(27, 28) It is recommended that people with RA get support to identify factors and means to manage symptoms like pain, fatigue, depression, anxiety, and sleep problems.(7, 10-12, 26, 29) Our study highlights that inadequate attention to aspects of the disease beyond medical treatment can leave patients feeling alone and disappointed. To address this, clarifying the roles of healthcare professionals and discussing expectations with patients might help ensure comprehensive support. It is important to recognize that these patients may benefit from additional healthcare contacts that focus on overall well-being. The unclear understanding of the physician's role might contribute to unnecessary dissatisfaction, emphasizing the need to clarify the different roles of healthcare professionals and their respective contributions. In addition, regarding the meeting with the rheumatologist and the patients' wish to be seen, a simple question about 'How has it been for you lately' might change the perception of the meeting. Although none of the respondents expressed they had received bad medical treatment, some respondents did experience a bad patient-physician interaction. This can be improved by very small changes, e.g. including interested responses on the patient's own experience and reflections could increase the quality of the meeting.

Another aim of this study was to explore opinions on the benefit of a digital self-care application in improving patient-doctor communication. Finding the most suitable treatment for persons with RA is challenging for both patients and healthcare personnel because of the chronic nature of the disease. Self-management using digital tools opens a new treatment era with the potential to better meet the needs and preferences of persons with RA, improve overall health outcomes, and increase satisfaction with their interaction with healthcare personnel.(30) As our respondents expressed that living with RA can be different from day to day depending on disease symptoms or treatment side effects. Individual with RA carries the burden of managing their daily life, which may also include coping with pain, fatigue, and medications, etc. In this regard, most of the activities to promote health in persons with RA occur outside of the rheumatologist's office.

Our respondents appreciated when the healthcare personnel took their observations into account when following up on disease activity. However, not all had a good experience of that, which was permeated with disappointment and surprise. Therefore, we would like to argue that strengthening self-management by using a digital tool to log on what happens between visiting healthcare should be promoted by the healthcare, not by the person with RA. From the healthcare perspective, digital tools for self-management may increase the efficiency and time spent during visits, improving relationships with patients, and using more precise data to guide treatment decisions.(6)

Different digital tools could be a solution to improve access to care in parts of the country that do not have teamwork for this patient group. It can also improve the timeliness of care, addressing urgent health issues quickly. It could also improve the efficacy of care by supporting improved outcomes with closer follow-up. That could include web-based education, videoconference, telephonically, or electronic messaging. This has been used successfully in oncology service and treatment,(31) but has also increased in healthcare overall during the COVID-19 pandemic.(32) Whether virtual-care modalities for RA can replace in-person rheumatologist visits while maintaining a comparable quality of care, has been investigated in a systematic review. They summarised the limited existing evidence regarding the impact of virtual rheumatology care in patients with RA on patient disease activity and patient experience and satisfaction with care. They found no difference in

For peer review only - http://bmjopen.bmj.com/site/about/guidelines.xhtml

observed outcomes between virtual care delivered by a rheumatologist and by a rheumatology nurse. However, virtual care was found to have additional benefits for improved treatment adherence, maintenance of functional status, and quality of life.(33) However, it is critical that those digital tools are evidence-based and focus on self-management interventions that are developed by healthcare providers and persons with RA to support and empower individuals.(34)

Strengths and limitations

The study focused on understanding the patient perspective on healthcare interaction through semi-structured interviews, specifically examining their opinions on the impact of a self-care application on patient-doctor communication. However, it should be noted that the findings may not be applicable to other disease groups, as they primarily reflect the experiences of this particular group at their specific disease stage. Additionally, a limitation of the study was the exclusion of participants who did not use the app, which could impact the generalizability of the results.

It is important to acknowledge the potential limitations of self-reporting bias and subjectivity in interview-based studies. Self-reporting bias can arise due to participants' inclination to provide socially desirable responses or selectively recall information. To mitigate this bias, we did our best to use strategies such as emphasizing the importance of honest and candid responses and ensuring confidentiality. Subjectivity is another limitation that needs to be addressed. We were aware of our own interpretations, biases, and preconceived notions that can influence data analysis and reporting. Engaging in reflexivity and maintaining reflexivity journals helped us to reflect on our own perspectives and potential biases. We were seeking input from peers or colleagues to provide valuable insights and alternative viewpoints. We employed an analytical frameworks or coding schemes in reputed dialog to enhance objectivity in data analysis.

For peer review only - http://bmjopen.bmj.com/site/about/guidelines.xhtml

Conclusion

In conclusion, the study highlights the need for healthcare providers, including physicians and nurses, to address areas where patient satisfaction may be lacking. It is essential to consider patient feedback and make improvements to enhance the quality of care provided. Suggestions for healthcare providers include actively listening to patient concerns, improving communication, and fostering a patient-centered approach. By addressing these areas of dissatisfaction, healthcare providers can strive to deliver a more satisfactory and patient-centered healthcare experience.

What we know is that technology is here to stay in our society and healthcare settings. Thus, more thinking needs to be done on how to best deploy digital tools effectively; specifically, how to balance digital meetings, physical meetings, patients' preparation, and what healthcare can provide regarding treatment. Our respondents' feelings about not being seen and being part of a dialog when they have logged their symptoms and prepared themselves might be due to an implementation gap between healthcare and the patients. Further, the next step might be to implement digital tools in the healthcare setting so that both the patient and the healthcare personnel have a shared understanding. Collaboration is essential in this context.

For peer review only

For peer review only - http://bmjopen.bmj.com/site/about/guidelines.xhtml

References

1. Conaghan PG, Green MJ, Emery P. Established rheumatoid arthritis. Best Practice & Research Clinical Rheumatology. 1999;13(4):561-75.
2. Smolen JS, Aletaha D, McInnes IB. Rheumatoid arthritis. Lancet. 2016;388(10055):2023-38.
3. Alamanos Y, Voulgari PV, Drosos AA, editors. Incidence and prevalence of rheumatoid arthritis, based on the 1987 American College of Rheumatology criteria: a systematic review. Seminars in arthritis and rheumatism; 2006: Elsevier.
4. Aletaha D, Neogi T, Silman AJ, Funovits J, Felson DT, Bingham CO, 3rd, et al. 2010 Rheumatoid arthritis classification criteria: an American College of Rheumatology/European League Against Rheumatism collaborative initiative. Arthritis Rheum. 2010;62(9):2569-81.
5. Majithia V, Geraci SA. Rheumatoid Arthritis: Diagnosis and Management. The American Journal of Medicine. 2007;120(11):936-9.
6. Mollard E, Michaud K. Self-Management of Rheumatoid Arthritis: Mobile Applications. Current Rheumatology Reports. 2020;23(1):2.
7. Smolen JS, Landewé RBM, Bijlsma JWJ, Burmester GR, Dougados M, Kerschbaumer A, et al. EULAR recommendations for the management of rheumatoid arthritis with synthetic and biological disease-modifying antirheumatic drugs: 2019 update. J Annals of the Rheumatic Diseases. 2020;79(6):685-99.
8. Treharne GJ, Lyons AC, Booth DA, Mason SR, Kitas GD. Reactions to disability in patients with early versus established rheumatoid arthritis. Scand J Rheumatol. 2004;33(1):30-8.
9. Gettings L. Psychological well-being in rheumatoid arthritis: a review of the literature. Musculoskeletal Care. 2010;8(2):99-106.
10. Larsson I, Bremander A, Andersson M. Patient Empowerment and Associations with Disease Activity and Pain-Related and Lifestyle Factors in Patients With Rheumatoid Arthritis. ACR Open Rheumatology. 2021;3(12):842-9.
11. Donnelly S, Manning M, Mannan H, Wilson AG, Kroll T. Renegotiating dimensions of the self: A systematic review and qualitative evidence synthesis of the lived experience of self-managing rheumatoid arthritis. Health Expect. 2020;23(6):1388-411.
12. Zuidema RM, Repping-Wuts H, Evers AWM, Van Gaal BGI, Van Achterberg T. What do we know about rheumatoid arthritis patients' support needs for self-management? A scoping review. International Journal of Nursing Studies. 2015;52(10):1617-24.
13. Perry B, Geoghegan C, Lin L, McGuire FH, Nido V, Grabert B, et al. Patient preferences for using mobile technologies in clinical trials. Contemporary Clinical Trials Communications. 2019;15:100399.
14. Nikiphorou E, Santos EJF, Marques A, Böhm P, Bijlsma JW, Daien CI, et al. 2021 EULAR recommendations for the implementation of self-management strategies in patients with inflammatory arthritis. J Annals of the Rheumatic Diseases. 2021;80(10):1278-85.
15. Bodenheimer T, Lorig K, Holman H, Grumbach K. Patient Self-management of Chronic Disease in Primary Care. JAMA. 2002;288(19):2469-75.
16. Von Korff M, Tiemens B. Individualized stepped care of chronic illness. The Western journal of medicine. 2000;172(2):133-7.
17. Kvale S, Brinkmann S. Interviews: Learning the craft of qualitative research interviewing: sage; 2009.
18. Krueger RA, Casey MA. Focus groups: A practical guide for applied research: Sage publications; 2014.

For peer review only - http://bmjopen.bmj.com/site/about/guidelines.xhtml

19. ATLAS.ti Scientific Software Development GmbH. ATLAS.ti Web - The easiest way to uncover qualitative insights 2022 [Available from: https://atlasti.com/atlas-ti-web.
20. Burnard P. Writing a qualitative research report. Nurs Educ Today. 2004;24(3):174-9.
21. Burnard P, Gill P, Stewart K, Treasure E, Chadwick B. Analysing and presenting qualitative data. Brit Dent J. 2008;204(8):429-32.
22. Hsiao B, Fraenkel L. Patient preferences for rheumatoid arthritis treatment. Curr Opin Rheumatol. 2019;31(3):256-63.
23. Bywall KS, Kihlbom U, Hansson M, Falahee M, Raza K, Baecklund E, et al. Patient preferences on rheumatoid arthritis second-line treatment: a discrete choice experiment of Swedish patients. Arthritis Res Ther. 2020;22(1):288.
24. Mathijssen EGE, Vriezekolk JE, Popa CD, van den Bemt BJF. Shared decision making in routine clinical care of patients with rheumatoid arthritis: an assessment of audio-recorded consultations. Ann Rheum Dis. 2020;79(2):170-5.
25. Nagy G, Roodenrijs NMT, Welsing PMJ, Kedves M, Hamar A, van der Goes MC, et al. EULAR points to consider for the management of difficult-to-treat rheumatoid arthritis. Ann Rheum Dis. 2022;81(1):20-33.
26. Hehir M, Carr M, Davis B, Radford S, Robertson L, Tipler S, et al. Nursing support at the onset of rheumatoid arthritis: Time and space for emotions, practicalities and self-management. Musculoskelet Care. 2008;6(2):124-34.
27. Radford S, Carr M, Hehir M, Davis B, Robertson L, Cockshott Z, et al. 'It's quite hard to grasp the enormity of it': Perceived needs of people upon diagnosis of rheumatoid arthritis. Musculoskelet Care. 2008;6(3):155-67.
28. Jones B, Bennett S, Larsson I, Zangi H, Boström C, Van der Elst K, et al. Disseminating and assessing implementation of the EULAR recommendations for patient education in inflammatory arthritis: a mixed-methods study with patients' perspectives. J RMD Open. 2022;8(1):e002256.
29. Knittle K, Maes S, de Gucht V. Psychological interventions for rheumatoid arthritis: Examining the role of self-regulation with a systematic review and meta-analysis of randomized controlled trials. Arthritis Care Res. 2010;62(10):1460-72.
30. Najm A, Lempp H, Gossec L, Berenbaum F, Nikiphorou E. Needs, Experiences, and Views of People With Rheumatic and Musculoskeletal Diseases on Self-Management Mobile Health Apps: Mixed Methods Study. JMIR Mhealth Uhealth. 2020;8(4):e14351.
31. Sirintrapun SJ, Lopez AM. Telemedicine in Cancer Care. 2018(38):540-5.
32. Wosik J, Fudim M, Cameron B, Gellad ZF, Cho A, Phinney D, et al. Telehealth transformation: COVID-19 and the rise of virtual care. Journal of the American Medical Informatics Association. 2020;27(6):957-62.
33. Han L, Hazlewood GS, Barnabe C, Barber CEH. Systematic Review of Outcomes and Patient Experience With Virtual Care in Rheumatoid Arthritis. Arthritis Care Res. 2022;74(9):1484-92.
34. Rodríguez Sánchez-Laulhé P, Luque-Romero LG, Barrero-García FJ, Biscarri-Carbonero Á, Blanquero J, Suero-Pineda A, et al. An Exercise and Educational and Self-management Program Delivered With a Smartphone App (CareHand) in Adults With Rheumatoid Arthritis of the Hands: Randomized Controlled Trial. JMIR Mhealth Uhealth. 2022;10(4):e35462.

For peer review only - http://bmjopen.bmj.com/site/about/guidelines.xhtml

Example of App Content: Explore snapshots of the app's features. For more details, please visit: https://www.elsa.science/en/

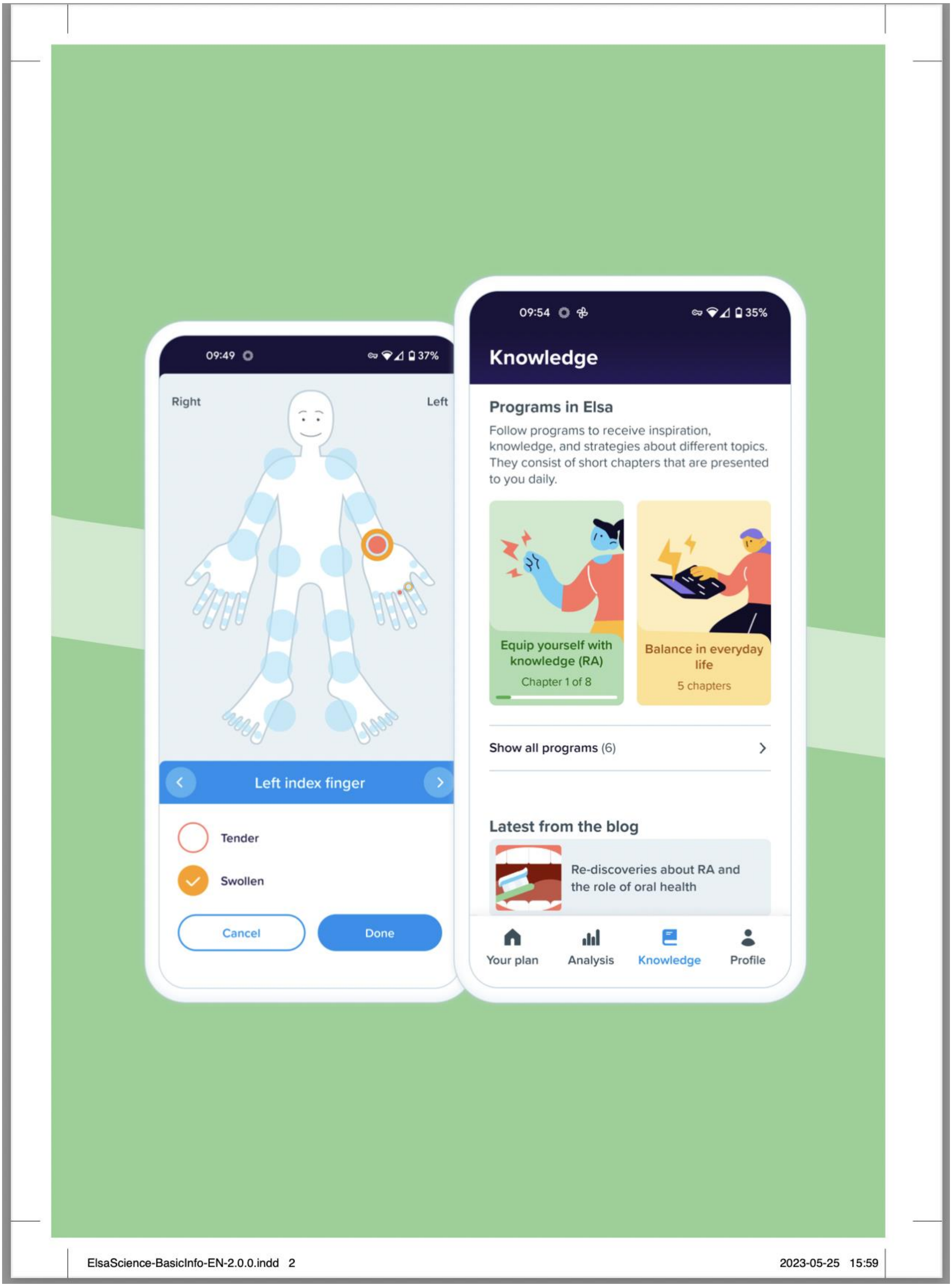

The Self-Care App

Rheumatic disease?
This is for you.

The Elsa Science Self-Care App is your partner in managing rheumatic conditions at any stage of the disease so you can focus on quality of life.

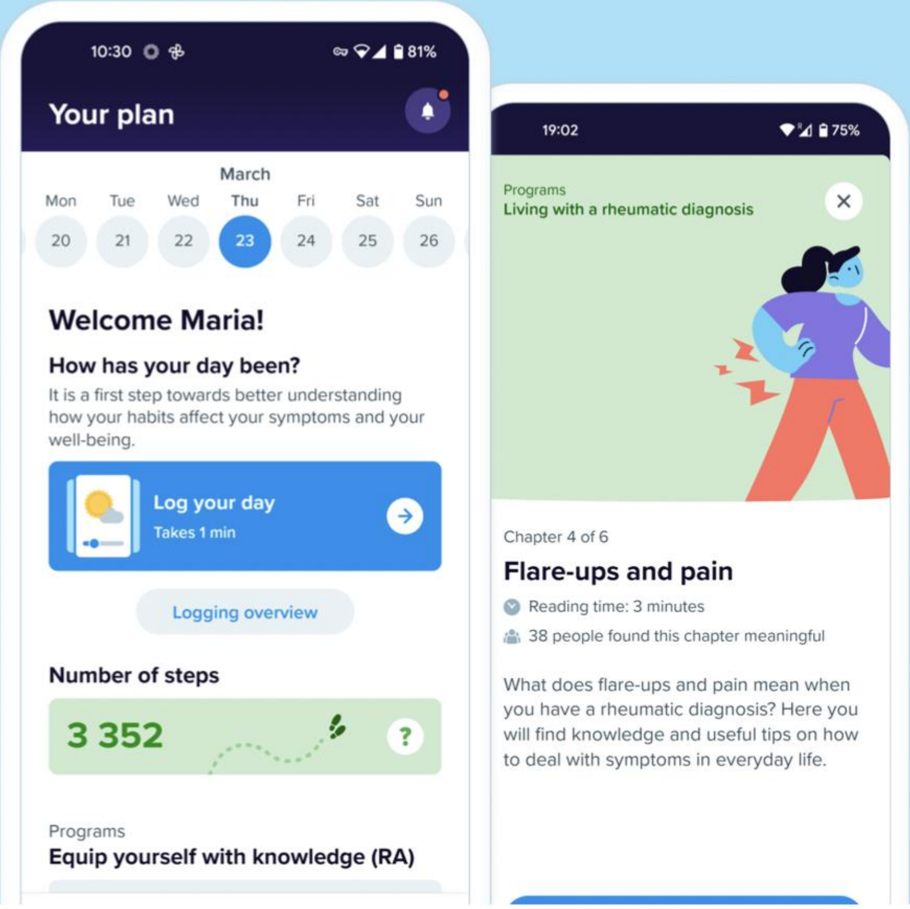

ElsaScience-BasicInfo-EN-2.0.0.indd 1 2023-05-25 15:59

COREQ (COnsolidated criteria for REporting Qualitative research) Checklist

A checklist of items that should be included in reports of qualitative research. You must report the page number in your manuscript where you consider each of the items listed in this checklist. If you have not included this information, either revise your manuscript accordingly before submitting or note N/A.

Topic	Item No.	Guide Questions/Description	Reported on Page No.
**Domain 1: Research team and reflexivity**			
Personal characteristics			
Interviewer/facilitator	1	Which author/s conducted the interview or focus group?	
Credentials	2	What were the researcher's credentials? E.g. PhD, MD	
Occupation	3	What was their occupation at the time of the study?	
Gender	4	Was the researcher male or female?	
Experience and training	5	What experience or training did the researcher have?	
Relationship with participants			
Relationship established	6	Was a relationship established prior to study commencement?	
Participant knowledge of the interviewer	7	What did the participants know about the researcher? e.g. personal goals, reasons for doing the research	
Interviewer characteristics	8	What characteristics were reported about the inter viewer/facilitator? e.g. Bias, assumptions, reasons and interests in the research topic	
**Domain 2: Study design**			
Theoretical framework			
Methodological orientation and Theory	9	What methodological orientation was stated to underpin the study? e.g. grounded theory, discourse analysis, ethnography, phenomenology, content analysis	
Participant selection			
Sampling	10	How were participants selected? e.g. purposive, convenience, consecutive, snowball	
Method of approach	11	How were participants approached? e.g. face-to-face, telephone, mail, email	
Sample size	12	How many participants were in the study?	
Non-participation	13	How many people refused to participate or dropped out? Reasons?	
Setting			
Setting of data collection	14	Where was the data collected? e.g. home, clinic, workplace	
Presence of non-participants	15	Was anyone else present besides the participants and researchers?	
Description of sample	16	What are the important characteristics of the sample? e.g. demographic data, date	
Data collection			
Interview guide	17	Were questions, prompts, guides provided by the authors? Was it pilot tested?	
Repeat interviews	18	Were repeat inter views carried out? If yes, how many?	
Audio/visual recording	19	Did the research use audio or visual recording to collect the data?	
Field notes	20	Were field notes made during and/or after the inter view or focus group?	
Duration	21	What was the duration of the inter views or focus group?	
Data saturation	22	Was data saturation discussed?	
Transcripts returned	23	Were transcripts returned to participants for comment and/or	

For peer review only

Topic	Item No.	Guide Questions/Description	Reported on Page No.
		correction?	
**Domain 3: analysis and findings**			
Data analysis			
Number of data coders	24	How many data coders coded the data?	
Description of the coding tree	25	Did authors provide a description of the coding tree?	
Derivation of themes	26	Were themes identified in advance or derived from the data?	
Software	27	What software, if applicable, was used to manage the data?	
Participant checking	28	Did participants provide feedback on the findings?	
Reporting			
Quotations presented	29	Were participant quotations presented to illustrate the themes/findings? Was each quotation identified? e.g. participant number	
Data and findings consistent	30	Was there consistency between the data presented and the findings?	
Clarity of major themes	31	Were major themes clearly presented in the findings?	
Clarity of minor themes	32	Is there a description of diverse cases or discussion of minor themes?	

Developed from: Tong A, Sainsbury P, Craig J. Consolidated criteria for reporting qualitative research (COREQ): a 32-item checklist for interviews and focus groups. *International Journal for Quality in Health Care*. 2007. Volume 19, Number 6: pp. 349 – 357

Once you have completed this checklist, please save a copy and upload it as part of your submission. DO NOT include this checklist as part of the main manuscript document. It must be uploaded as a separate file.

For peer review only

BMJ Open

Experiences of individuals with rheumatoid arthritis interacting with health care and the use of a digital self-care application: a qualitative interview study

Journal:	*BMJ Open*
Manuscript ID	bmjopen-2023-072274.R2
Article Type:	Original research
Date Submitted by the Author:	10-Oct-2023
Complete List of Authors:	Viberg Johansson, Jennifer; Uppsala University, Department of Public Health and Caring Sciences<br>Blyckert, Hanna; Elsa Science<br>Schölin Bywall, , Karin; School of Health, Care and Social Welfare, Division of Health and Welfare Technology; Uppsala University Department of Public Health and Caring Sciences, Centre for Research Ethics & Bioethics
**Primary Subject Heading**:	Communication
Secondary Subject Heading:	Ethics, Health services research, Rheumatology
Keywords:	Rheumatology < INTERNAL MEDICINE, Self Care, Patient Participation

SCHOLARONE™
Manuscripts

I, the Submitting Author has the right to grant and does grant on behalf of all authors of the Work (as defined in the below author licence), an exclusive licence and/or a non-exclusive licence for contributions from authors who are: i) UK Crown employees; ii) where BMJ has agreed a CC-BY licence shall apply, and/or iii) in accordance with the terms applicable for US Federal Government officers or employees acting as part of their official duties; on a worldwide, perpetual, irrevocable, royalty-free basis to BMJ Publishing Group Ltd ("BMJ") its licensees and where the relevant Journal is co-owned by BMJ to the co-owners of the Journal, to publish the Work in this journal and any other BMJ products and to exploit all rights, as set out in our licence.

The Submitting Author accepts and understands that any supply made under these terms is made by BMJ to the Submitting Author unless you are acting as an employee on behalf of your employer or a postgraduate student of an affiliated institution which is paying any applicable article publishing charge ("APC") for Open Access articles. Where the Submitting Author wishes to make the Work available on an Open Access basis (and intends to pay the relevant APC), the terms of reuse of such Open Access shall be governed by a Creative Commons licence – details of these licences and which Creative Commons licence will apply to this Work are set out in our licence referred to above.

Other than as permitted in any relevant BMJ Author's Self Archiving Policies, I confirm this Work has not been accepted for publication elsewhere, is not being considered for publication elsewhere and does not duplicate material already published. I confirm all authors consent to publication of this Work and authorise the granting of this licence.

For peer review only

Experiences of individuals with rheumatoid arthritis interacting with health care and the use of a digital self-care application: a qualitative interview study

Jennifer Viberg Johansson, Hanna Blyckert, Karin Schölin Bywall

Jennifer Viberg Johansson (she/her)
Department of Public Health and Caring Sciences, Centre for Research Ethics & Bioethics, Uppsala University, Uppsala, Sweden
jennifer.viberg-johansson@crb.uu.se

Hanna Blyckert (she/her), B.Sc.
Elsa Science, Stockholm, Sweden
hanna@elsa.science

Karin Schölin Bywall (she/her), Ph.D.
School of Health, Care and Social Welfare, Division of Health and Welfare Technology, Mälardalen University, Västerås, Sweden
&
Department of Public Health and Caring Sciences, Centre for Research Ethics & Bioethics, Uppsala University, Uppsala, Sweden
karin.scholin.bywall@mdu.se

Correspondence to:
Jennifer Viberg Johansson
jennifer.viberg-johansson@crb.uu.se

Keywords: self-management, mobile application, patient perspectives, patient preferences, pedagogic program, rheumatoid arthritis

Word count: 6244

For peer review only

Abstract

Objectives: Over the last few decades, there have been significant improvements in the treatment of rheumatoid arthritis (RA), with the development of new treatments and guidelines for teamwork and patient self-care, and access to digital tools. This study aimed to explore the experiences of individuals with RA interacting with healthcare. It also looked at how a self-care application, an educational program called the "Healthcare Encounter", improved patient-doctor communication.

Design: Semi-structured interviews were conducted, and qualitative content analysis was performed.

Setting: The potential participants, individuals with established, or under investigation for, RA diagnosis at rheumatology clinics in Sweden, were asked to participate in the study via a digital self-care application called the Elsa Science Self-care app.

Participants: Ten interviews were performed with participants from nine clinics following a meeting with the rheumatologist or other healthcare personnel between September 2022 and October 2022. Phrases, sentences, or paragraphs referring to experiences from healthcare meetings and opinions about the digital program were identified and coded. Codes that reflected similar concepts were grouped; sub-categories were formulated, and categories were connected to their experiences and opinions.

Results: Among our participants, three main categories emerged: the availability of healthcare, individual efforts to have a healthier life, and personal interaction with healthcare. Participants described that the "Healthcare Encounter" educational program can be a source of information, which confirms, supports, and creates a sense of control.

Conclusion: The participants valued being seen and taking part in a dialog when they had prepared themselves (observed symptoms over time and prepared questions). The implementation of digital self-care applications might need to be incorporated into the healthcare setting so that both the patients and the healthcare personnel have a shared understanding. Collaboration is essential in this context.

Strengths and limitations of this study

- The study employed semi-structured interviews to delve into patients' perspectives on interacting with healthcare, allowing for a comprehensive exploration of their experiences.
- An in-depth investigation was conducted of patients' opinions on the impact of a self-care application on patient-doctor communication, shedding light on the effectiveness and potential improvements in this crucial aspect of healthcare.
- The study findings may not be transferable to other disease groups, as the study investigated how this particular group at this disease stage experienced healthcare.
- Another limitation of the study was that only participants who used the app were invited to participate.

For peer review only - http://bmjopen.bmj.com/site/about/guidelines.xhtml

For peer review only

For peer review only - http://bmjopen.bmj.com/site/about/guidelines.xhtml

INTRODUCTION

Rheumatoid arthritis (RA) is a chronic autoimmune disease, causing joint pain and inflammation (1). It affects about 0.5–1% of the population (2, 3), commonly appearing in joints such as the hands, knees, and ankles, often symmetrically. RA can start gradually over weeks or suddenly overnight. Early symptoms include fatigue, malaise, and swelling in the small joints, often with morning stiffness lasting over an hour. Moreover, it can lead to complications in various organs (4, 5).

There has been significant progress in RA treatment in recent decades, including new medications, teamwork guidelines, self-care promotion, and digital tool accessibility (6). While there is no cure, medications can relieve symptoms and preserve joint function. Combining treatments for optimal symptom relief often requires adjustments due to new symptoms or side effects. RA care should include patient education and effective communication to empower patients and promote shared decision-making (7).

In addition to the physical challenges of RA, individuals also experience psychological struggles, such as distress and helplessness (7). They constantly feel unwell, which can restrict their engagement in daily life and social activities. Moreover, psychological well-being, including self-esteem, body image, and relationships, is negatively affected by joint tenderness in individuals with RA (8). Besides finding the right medication use and hindering the development of disabilities in the joints, guidelines recommend providing education and tools to handle the psychological aspects of the disease (4). There are recommendations to help patients identify factors and means to manage symptoms such as pain, fatigue, depression, anxiety, and sleep problems (9). Earlier research has emphasized the need for education in self-management skills and counseling to enhance empowerment and improve the emotional dimension of care. Findings support the need for team-based rehabilitation interventions to enhance empowerment in patients with RA (10, 11). Moreover, results from a review of RA patients' self-management support needs show that individuals with RA have informational, emotional, social, and practical needs. Informational needs can be about how to deal with pain, exercise, and medication. Emotional support can be from their relatives or healthcare professional (12).

Using mobile technology as a complement in healthcare has increased during the last decade and is now a natural part of healthcare-related services in general (13, 14), as well as for individuals with RA who require daily self-management of the disease (6). The adoption of digital tools has transformed healthcare, offering RA patients access to reliable information, self-tracking, and self-management outside the hospital setting. These tools empower patients to prepare for healthcare interactions. Additionally, they have proven to be more effective and cost-efficient in treating RA (15). When comparing traditional care with collaborative care, it is evident that there is a need for a shared understanding between RA patients and healthcare professionals.

Both approaches emphasize patients' responsibility for their health, but healthcare providers play a vital role in communicating, motivating, and aiding patients in self-management (16). Medical evidence, guidelines, and digital technologies are in place for quality RA care. The question is whether Sweden's healthcare system is prepared for more active patient involvement and effective interactions between RA patients and rheumatologists. There is a

For peer review only - http://bmjopen.bmj.com/site/about/guidelines.xhtml

need to investigate RA patients' experiences of healthcare interactions and the use of digital tools for self-care and communication.

This study aimed to explore the experiences of individuals with RA, or under investigation for RA, interacting with healthcare. It also aimed to determine how helpful a digital self-care application was in improving patient-doctor communication.

For peer review only

For peer review only - http://bmjopen.bmj.com/site/about/guidelines.xhtml

METHODS

Design

The was a qualitative study based on semi-structured interviews.

Setting and participants

The potential participants, individuals with established, or under investigation for, RA diagnosis (Swedish guidelines for diagnosis), at rheumatology clinics in Sweden, were asked to participate in the study via a digital self-care application called Elsa (https://www.elsa.science/en/; **supplementary file**). The app was accessible through the Google Play Store for Android and the App Store for iOS. The participants discovered it through various channels, including recommendations from clinic staff or brochures at health centers, digital platforms and social media, or through their own search for self-care. Participants were eligible for the survey if they had an RA diagnosis, were aged 18–80-years-old, and understood and expressed themselves in Swedish. The structure of the study was to first participate in a survey, then perform a specific program in the Elsa app called the "Healthcare Encounter" and, in conclusion, complete a final survey. At the time of the final survey, they could choose to sign up for a follow-up interview.

The specific program aims to provide basic knowledge to individuals living with a rheumatic disease that can inspire them to make sustainable lifestyle changes and improve their well-being. The "Healthcare Encounter" program focuses on providing basic knowledge about the treatment, medical options, what to expect from meeting healthcare, and how the individuals themselves can prepare before the meeting. This educational program takes about 20 minutes to complete. Each person could decide for themselves whether they would like to do it all at once or do parts of it over time. It takes about 2-3 minutes per day to fill in the daily log. See Box 1 for a summary of the content of the educational program.

Box 1. Content of the educational program

- Introduction, background, and basic knowledge
- The goal of the encounter (treatment goals)
- The treatment in general (different medication options)
- Long-term collaboration (the healthcare system's role and your role as a patient)
- How to prepare (before the encounter)
- How to act during the encounter
- How to follow up after the encounter
- Checklist

Data collection

Eighteen out of 43 individuals declared their interest in participating in this interview study. The participants were then asked via e-mail to schedule an interview. The study included participants who had appointments scheduled within the near future or within the past month. Ten interviews were performed after the meeting with the rheumatologist or other healthcare personnel from nine different clinics in Sweden between September 2022 and October 2022 by the second author (HB).

The interviews lasted 44 to 63 minutes and were conducted in Swedish via Google Meets or telephone (n=1). We began each interview by asking the participants about their experience

For peer review only - http://bmjopen.bmj.com/site/about/guidelines.xhtml

of having RA, their treatment, and health in general. Thereafter, we asked them to describe their experience before, during, and after the healthcare meeting. They were also asked to describe their use of the digital program, called the “healthcare encounter”, regarding whether it was useful to them or if it was missing any information or function. A semi-structured interview guide with open-ended questions (17, 18) was developed by the authors (Table 1). The interview guide was pilot tested among colleagues and an individual with RA to assess the comprehension of the questions. The sequence of questions was reorganized to align with the temporal aspects of preparations and experiences before, during, and after the medical appointment and the use of the app.

Table 1. Interview guide used for the semi-structured interviews

Description of the project, the goal, and our background knowledge and interest.<br>Can you please tell me about yourself and your life at the moment (warm-up question)
Before your appointment with the healthcare provider – how did you feel before your visit?<br>Probing questions: Did you prepare in any way? If so, how? Was there anything specific you planned to bring up at the meeting? Did you get any help from the program in your preparation? If so, what? Was there any difference in how you prepared now compared to how you usually prepare yourself?
During the appointment with the healthcare provider – how was the visit?<br>Probing questions: What did you talk about (feeling in general, treatment, side effects, etc.)? What felt good/less good during the meeting? Did you have the opportunity to bring up what you wanted to talk about (treatment options, any side effects, other complaints, what is important to you)? Compared to previous visits, how did you experience this?
After the appointment with the healthcare provider – do you remember how you felt after the visit?<br>Probing questions: Was the meeting as you wanted it to be? Would you like to change anything if you could, in retrospect? In retrospect, is there anything that feels unclear after the meeting (missing info, unsure of how to do something, proceeding with self-care)? How have you handled the uncertainty?
About the program – what do you think about the program?<br>Probing questions: Was there something that you liked in particular? Something you liked less or even disliked? Is there something you missed or would like more of?

Analysis

The recorded interviews were transcribed verbatim by a professional transcription company. Thereafter, we listened to the recordings in their entirety to verify the transcriptions. After all the transcripts were read again, meaning units (phrases, sentences, or paragraphs), with experience about healthcare meetings and opinions about the digital program, were identified for further scrutiny. The material contained 344 meaning units in total. Atlas.ti Web (19) and Microsoft Excel (2016) were used to assist in the data management and analysis process. In the next stage of the process, we continued with comparisons of the meaning units, examining their similarities and differences from the perspective of experiences and healthcare options and the digital program. Open coding of each meaning unit was added, which summed up what was being said in the text. Two of the interviews were coded simultaneously by both authors (JVJ and HB), who jointly discussed what meaning units to identify as well as interpretations and formulations of the codes, establishing the initial coding framework. The remaining interviews were coded by JVJ. However, when new codes emerged, digital meetings were held with all co-authors to discuss the integration into the existing scheme. Codes that reflected a similar concept were grouped; sub-categories were formulated, and categories were identified (20, 21) by JVJ and thereafter discussed thoroughly with HB and KSB (Table 2). Additionally, two meetings were held with the greater project’s research group (NORA) to solicit feedback on the drafted results of both the main categories and the sub-categories, accompanied by illustrative quotations. Thematic

For peer review only - http://bmjopen.bmj.com/site/about/guidelines.xhtml

saturation was reached regarding the aim of the data collection. The authors, JVJ, HS, and KSB, bring several years of experience in conducting interviews and analyzing qualitative data. Field notes were taken both during the interviews and throughout the analysis process.

Table 2. Example of the analytical process of the experiences when interacting with the healthcare providers

Meaning unit	Initial coding	Sub-category	Category
But like [trying to get in touch], you just feel, what the hell, I have no power.	Does not reach the healthcare providers	Accessibility and confirmation of care	Availability of healthcare providers
I have expressed several times to them on the phone, I feel alone. I feel very alone. I think it is hard actually. But then, it's not like I'm lying down and crying about it, but I feel left out.	Feeling alone and left out	To be taken care of	Availability of healthcare providers
He is responsive, he listens, understanding […] it is a pleasure to go and see him. I never feel any stress or pressure when I talk to him. He is very responsive and listens... he always takes what I say seriously.	He is responsive, listens, and explains; he takes what I say seriously.	To be seen and met with interest	Personal interaction

Patient and public involvement

None.

For peer review only

RESULTS

In total, 10 interviews (seven females, three males) were conducted with persons with, or under investigation for, RA (n=1 under investigation with symptoms two years back in time and then later, after the interview, the person received a confirmed diagnosis of RA). Disease duration ranged from one to three years, with one exception, where the person received a diagnosis 12 years ago. The participants were aged 45–76-years-old (mean age 56.7) and were from different demographic locations in Sweden. All of them were taking medication, and they were recruited from across the entire country. Moreover, all participants were accustomed to using smartphones and mobile applications in their daily lives. They all described their experience of being diagnosed with RA as a challenge. Those who were still unsure of what their symptoms indicated expressed frustration about not knowing or understanding their body's signals. They had tried various healthcare services that might be helpful, and also medications that might work. Having problems linked to other diseases was also described as a challenge. Consequently, they described having to choose which health issue to alleviate first. Accepting the disease was experienced as both a challenge and a necessity. What was also described as a challenge, and a necessity, was finding a balance in life between activity and recovery, work and leisure, and physical activity and rest.

The next section describes the participants' experiences of interacting with healthcare providers and the use of the program, divided into three main categories. Thereafter, nine sub-categories were used to classify the discussions. The categories were: 1) Availability of healthcare, 2) Individual effort, and 3) Personal interaction. An overview of the categories and sub-categories is presented in Table 3. Below, these categories and sub-categories are described and illustrated using quotes.

Table 3. Categories and sub-categories of participants' experiences when interacting with healthcare providers

**Category**	**Sub-category**
Availability of healthcare	To feel prioritized
	Accessibility and confirmation of care
	To be taken care of
Individual effort	Taking care of myself
	Opportunity to equip myself
Personal interaction	To be seen and met with interest
	Gain self-efficacy
	Have a dialogue
	Met with competence

Availability of healthcare

The category *Availability of healthcare* was expressed in three different ways: *To feel prioritized*, *Accessibility and confirmation of care*, and *To be taken care of.*

To feel prioritized

This sub-category focuses on the perception of time availability for patients and healthcare personnel to discuss symptoms, treatment options, and resource allocation. About half of the participants felt prioritized and supported by their healthcare team, receiving excellent care.

For peer review only - http://bmjopen.bmj.com/site/about/guidelines.xhtml

However, the other half reported feeling neglected during their interactions with healthcare providers. They found care meetings to be too short, lacking sufficient time to discuss matters related to their disease beyond medication, and felt unable to ask follow-up questions. The time constraint hindered the establishment of a personal connection. Some even felt de-humanized, i.e. treated like they were part of an assembly line rather than as individuals.

> *Well, I would have liked his [the rheumatologist] clinical part first[...] go through the joints and any blood tests and explain what the result means, so that you might learn something yourself. So you don't have to keep asking more times. And I wish him to ask, 'How does everyday life work for you?' I still have not been asked that question since 2019.* (Respondent 3)

Some participants suggested the benefit of having more frequent meetings early on in the process, especially when more issues arise and there are concerns. The possibility of having additional meetings, if necessary, gives the impression that you are valued. Meeting healthcare personnel who are unfamiliar with rheumatic disease can lead to feelings of insecurity, creating a perception that this patient group is not given priority.

> *..then I felt some insecurity. If it were someone who had the actual expertise, then they may have asked other follow-up questions. The person was very nice, but it felt unsafe. It felt like, who is taking care of me? It's not her fault, of course, but that's how I felt...*(Respondent 7)

Accessibility and confirmation of care

Having healthcare that is accessible is greatly appreciated and valuable for the participants. This sub-category focuses on access, contact, and knowing where to seek assistance. Some expressed that it was easy to make contact via phone or messages via the digital platform used in Sweden (named 1177). When problems arise with symptoms or side effects, fast response, action, and reply are appreciated, giving a calming feeling. Having regular meetings scheduled also led to feeling safe.

> *I think it [meeting the rheumatologist] feels safe. I think it feels great. So, I'm happy to continue with that.* (Respondent 5)

However, not all participants experienced this. Most found it difficult to access healthcare through phone calls or messages. One respondent described it as a 'whispering game,' where they would call and relay the problem to a healthcare personnel, who would then forward the message to the rheumatologist. The rheumatologist would then send a message back through a third person. These challenges in reaching healthcare services are perceived as unsafe care, leading to a feeling of being exposed to danger.

> *...and I know that healthcare is generally heavily burdened, so without talking badly about an individual. But like, you just feel, what the hell, I have no power.* (Respondent 7)

The fact that the healthcare services provide a confirmation is also appreciated and valuable to the participants. Some described that the rheumatology clinic does a fantastic job and that you, as a patient, get quick answers if you have concerns or experience practical obstacles with medications. They also stated they receive a good follow-up of blood test results. However, for other participants, it can take a long time to get confirmation of test results.

For peer review only - http://bmjopen.bmj.com/site/about/guidelines.xhtml

Sometimes it is not until the next appointment or if they have indicated they are not doing well.

The participants who experienced collaboration regarding the treatment of RA expressed that the various healthcare professions had good accessibility and followed up on their work, and it was clear how to reach out to them when a problem occurred.

> *I have to say that I am very impressed with the rheumatologist at the hospital [...] and I do that [send a message] and it can take within an hour then I've got a response that either the nurse has answered, or she says that 'I've passed it on to your doctor' and then in the afternoon, I get an answer. So, this is how it has been for these four years, fantastic.* (Respondent 5)

To be taken care of

Participants in the study appreciated healthcare personnel who made them feel taken care of; they expressed gratitude for the teamwork they witnessed. Moreover, they found that receiving the right support gave them a sense of trust and reassurance during the difficult times. The use of a digital self-care application was seen as affirming their experiences and creating a feeling of being seen. Participants were positive about receiving answers to their questions and concerns via email or chat, as it allowed them to digest new information at their own pace. However, one respondent felt that the application's content did not reflect the reality of healthcare and described it as "too good to be true."

> *Other individuals' stories in the app, [...] I just… Yeah, but damn. That's me!* (Respondent 4)

A noble example, shared by one of the participants, was when the healthcare professional helped to refer her onward, instead of letting the patient seek help again on her own. Some of the participants experienced great teamwork and expressed pride in their care unit, stating that the staff had a nice and welcoming attitude, and the feeling of being looked after was strong.

> *…then he [the rheumatologist] had been talking to the gastroenterologist and discussed with them and put together an action plan with them.* (Respondent 6)

Some participants expressed frustration and insecurity due to a lack of follow-up and communication from the healthcare services regarding their symptoms, tests, and medication. Not hearing from healthcare services made them feel that they were not being cared for. Some participants believed that they had to time their appointments to be sick enough to receive the necessary help and felt that their symptoms were not taken seriously. Others struggled to find the right help and experienced difficulties in navigating the healthcare system, with different units not communicating with each other. Some participants had to take on the role of coordinating their own care, which was tiring and challenging. Finding the right care and prioritizing symptoms required significant effort and energy. In complex situations, such as having multiple symptoms and other diseases, participants struggled to find the appropriate care. Some considered changing to a larger hospital for better care.

For peer review only - http://bmjopen.bmj.com/site/about/guidelines.xhtml

Overall, there was a lack of clarity in knowing where to seek help within the healthcare system, resulting in frustration and being sent around to different units.

> *You are sent around a lot between the different healthcare units, depending on what problems arise. And there is no connecting link. One part of healthcare does not know what the other part is doing.* (Respondent 6)

Individual effort

The category of *Individual effort* was expressed in two different ways: *Taking care of myself* and having the *opportunity to equip myself.*

Taking care of myself

All participants expressed that they have tried to adapt to life in different ways, describing different attempts at self-care: adjusting their diet, trying different forms of exercise and training, and trying to find a balance between rest and activity. The participants described how they prepared themselves in different ways before the appointment with the rheumatologist. In addition to taking notes on how they feel – either with a pen and paper or via the digital tool – they sought information in different ways in order to equip themselves with knowledge. Some stated that equipping themselves with new knowledge was for their own sake. Others stated that it was also to facilitate healthcare, so they can be involved in the 'detective work' of finding effective treatment. Some expressed frustration that they must know a lot themselves to get help, that you must stand up for yourself to get good help, and that it feels like one has to be healthy to be able to be sick.

> *I don't think it's okay. I told her […] all the side effects. Then it was like 'Well, okay, let's try something else.' I have had to argue a lot to change my medication…* (Respondent 5)

Moreover, they try to understand and interpret their test results on the health and medical care's digital platform (name 1177) before the meeting. Many participants appreciated the application tool as a trustworthy source of information with a good spirit.

> *So, I google... being critical of sources, it's not that easy as a consumer. This [the application] feels very serious and here, they have talked to individuals who [are knowledgeable and experienced] ... that's the image I have, anyway.* (Respondent 7)

They valued being able to learn more about: what to expect with the disease, the medication, responsibilities of the different healthcare personnel, how to inform relatives, and what activities to engage in to feel better. Learning from others' experiences was also appreciated. One individual expressed that the self-care app could be useful even if one was not yet diagnosed.

Writing down questions, symptoms, and how they have felt over a longer period was a common action. For some, this was useful as a tool to reflect on their health, understand the reasons for the symptoms, and how they come and go, but also to remember the ups and downs of their symptoms.

> *I had a long list I had written before the phone meeting, where I tried to think 'How long have I been in pain?' 'Where do I have pain?' 'How do I*

For peer review only - http://bmjopen.bmj.com/site/about/guidelines.xhtml

> *react?' and 'What medicines have I taken?' and so on. So that I don't forget anything when I talk to the physician.* (Respondent 6)

The participants thought the self-care app was fun, helpful, and a way to take care of oneself when filling in the daily log. For some, it gave them time to reflect on how they feel and how the symptoms have changed over time. Some thought it would be helpful to keep a log on long-term pain, diet, physical activity, general mood, and sleeping pattern. They also expressed a need to be more specific in describing the exact location of the pain (e.g., where in the hand). Moreover, they suggested making a note of the possible reason for the pain by writing down the activity they were performing before the pain occurred. On the other hand, some participants said they forgot or felt they did not want to log their symptoms when everything was fine.

> *I usually go in and use it [logging symptoms], although it's become a bit more sporadic now the last... well, last month and so. Because I have felt better and then when I feel better, I kind of forget to fill it in.* (Respondent 9)

Opportunity to equip myself

There was a great variation in the extent to which our participants had the opportunity to be educated by their rheumatology clinic regarding their disease. Some had four days of education, some had one day or even half a day, and some did not receive any education. To receive education about the disease, medication, exercise, lifestyle habits, and how to find a balance in life was much appreciated by the participants who experienced these aspects at their clinic. They felt it made them feel safe and satisfied, even though life had changed with the diagnosis. Education was perceived to empower them and made it possible to take care of themselves.

> *... the introductory education... I mean, four full days. And so, they have it regularly [...] with everyone who is newly diagnosed, where they go through [everything].* (Respondent 6)

Those who did not receive education from the rheumatology clinic expressed a desire for it and hoped and even expected the healthcare services to provide it. Some described with surprise and disappointment that they had not received it. Understanding one's illness is deemed important; one wants to understand and take power over one's life. Specific aspects for which they wanted more knowledge were medical treatment, side effects, dietary suggestions, diagnosis, and what to expect from life.

> *...training and such, they talk about that and what is good. That you shouldn't smoke and like... yes, and so on. But diet, in particular, seems to be taboo.* (Respondent 8)

The digital self-care application was perceived as a good tool to help prepare before the meeting with the healthcare providers. Specifically, it increased their self-esteem, helping them to believe in themselves. After going through the program, participants created a list of good things to prepare prior to the healthcare meetings to be helpful. The list helped them to focus on the most important parts and not forget what is important. Looking back on their personal log was also appreciated as a memory support tool. Some participants suggested that the digital self-care application should promote healthy lifestyle choices and include personal

For peer review only - http://bmjopen.bmj.com/site/about/guidelines.xhtml

goals and interim goals. The inclusion of information and educational material for loved ones or acquaintances was seen as positive, as it can ease the burden and foster good relations among individuals' support network. The function of logging symptoms was seen as a good basis for a discussion with the rheumatologist. Some participants wished that the healthcare services had access to their logged in data to improve conversations and treatment options. However, some felt that constantly being reminded of their illness through the log can be burdensome.

> *…there is a risk that going on and on like that [with reading and log symptoms every day]… that you dig into your illnesses, and I don't think that feels very good.* (Respondent 2)

Personal interaction

Finally, our analysis revealed experiences related to personal interaction, and how the healthcare professionals' attitudes made the participants feel. This is the most comprehensive category regarding the participants' narratives. This category was expressed in four different ways: *To be seen* and *met with interest*, *Have a dialogue*, *Gain self-efficacy*, and *Met with competence*.

To be seen and met with interest

There was a strong emphasis on the appreciation of being seen, namely that professionals would show empathy towards the patient. The participants focused on the personal interaction with the physician and whether one feels that he or she is being attended to individually. They wanted to feel as though they were believed and to gain some hope in difficult situations, and not perceived as they were whining. Moreover, they wanted to be seen as a whole individual and talked to as an equal and not have the personnel look down on them. When the doctor takes the time and listens, the feeling of being seen as a person increases.

> *Very professional, very calm, and nice and she gave me hope.* (Respondent 5)

Some expressed feeling frustrated, stupid, and disregarded by rheumatologists who did not listen to their concerns or consider their perspective. They believed that a narrow focus on medication and side effects, without addressing other aspects of the disease, was unhelpful. Patients valued rheumatologists who took the time to listen, showed professionalism, and respected their needs. However, if healthcare professionals did not fulfill their promises or pay attention to their medical records, patients felt disregarded. They appreciated rheumatologists, who were knowledgeable and prioritized the need to discuss relevant topics during the appointment. They believed that a collaborative approach, where both the doctor and the patient shared their expertise, improved the encounter. Patients felt more comfortable asking important questions when the rheumatologist took their time and listened to them. Some participants described feeling like just a body to be examined by the rheumatologists and felt unable to discuss their specific pain and symptoms. One participant felt that her medical history was uninteresting to the rheumatologist, while another admitted to lying about her pain levels to ensure her complaints were taken seriously. Patients wanted the

For peer review only - http://bmjopen.bmj.com/site/about/guidelines.xhtml

physician to consider their lived experience of symptoms and side effects during the treatment discussions.

> *... they don't know me. I've met him once, and yet, they somehow assume that… you need to bite the bullet. It's a bit like that. And the first time ever when I was at the health center, when my fingers hurt... and then I had red knuckles. They were really inflamed. Then the medical center doctor told me that I have to learn to live with that; you just have to bite the bullet.* (Respondent 8)

Have a dialogue

The participants appreciated having a good dialog with the rheumatologist; being able to reason with the rheumatologist about medical treatments and side effects and finding a balance between things in life were perceived as valuable. Moreover, the participants appreciated the rheumatologist taking advantage of the preparation done by the patient before the meeting. Being able to reason about the amount of medication was also appreciated.

Those who did not have the opportunity to have a good dialog expressed sadness or frustration about what was missing for them. They all described they had heard that other patients had received better care. Lack of a good dialog caused the participants to feel they could not trust the rheumatologist; thus, they either wanted to change doctors or choose their own paths when it came to medication or lifestyle changes. For some, it was not clear whether it was ok to be critical or even change their rheumatologist if the communication did not work out well; there was a fear that it might result in bad consequences.

> *So, actually, I would like to change my physician. Because there is another doctor who helped me at some point when the joints in my hand were painful. He was very nice, accommodating and calm, and such. So actually, I want him. But then you don't know if you dare to change to another physician like that, because then [...] what if I get the evil eye […]. So, I feel, it's better I go there to those meetings with him, and he does what he has to do, then I google and learn by myself.* (Respondent 3)

There was a feeling of resignation among those who had prepared beforehand but were not listened to. Even though they prepared well (what to ask and written down how they have felt during the past month), the rheumatologist did not take notice of it. They shared that the doctor did not even look them in the eyes; was not interested in the patient's notes or questions; and answered using difficult language. Instead, the rheumatologist proceeded with a clinical test and made some comments about the medication. These participants felt they were being ignored, while they could instead have been a valuable resource.

> *[…] even though I had kept a log for a very long time, there was nothing that he [the rheumatologist] cared about when I was there. So, he looked there and then, the day I was there: 'Are you in pain here, yes. Are you not in pain here, no'? And then it was fine with that.* (Respondent 8)

Gain self-efficacy

Something that was expressed by all participants was the need for encouragement and hope for the future. Healthcare was seen as an important component in their pursuit of a life that

For peer review only - http://bmjopen.bmj.com/site/about/guidelines.xhtml

was functional and of quality. It is important to be able to express yourself and be taken seriously; then, there is a feeling of being competent in dealing with the new situation. For some, seeking healthcare services can often create a feeling that one has become old and soon dying. Getting help to set new goals and sub-goals was perceived as helpful; it helps to believe in the future.

> *… I have thought about it quite a lot and that helps me, that I need to have a goal. Not a dream, a goal. Everyone has dreams, but then you can pick certain dreams and say that is a dream, and this is my new goal. The goal for me is to get back on the police motorcycle. And being able to function and not have to think about how I'm paddling a kayak with my wrist or how I'm holding the weight or the dumbbell, or whatever it is. But I should just be able to be, as I was before.* (Respondent 3)

Met with competence

If the rheumatologist is knowledgeable and competent, it infuses confidence in the patient. Some participants are impressed by the rheumatologist's skills in balancing the trade-off between deploying different medications at different times. Those participants felt that they received all the answers they needed to remain calm and reassured, and following up on any side effects or symptoms was appreciated.

> *…questions about the medicine to the rheumatologist... she is good at explaining.* (Respondent 4)

Two participants felt they received better care if they were prepared and knew about things themselves. Some participants, however, were disappointed as they did not receive answers to their questions and felt the rheumatologist was only guessing when responding to them, resulting in them feeling resigned.

> *No, but he seems not to care; it feels so stiff when I talk to him. I had to remind him […]. Well, he's not well-read and that's what annoys me so much when he calls*. (Respondent 10)

For peer review only

For peer review only - http://bmjopen.bmj.com/site/about/guidelines.xhtml

DISCUSSION

This study emphasizes the importance of patient-centered care for individuals with RA. It highlights the significance of considering patients' psychological well-being, together with their physical health. This is in line with the EULAR recommendations and earlier research (7, 22). The study found that patients valued time and an open dialogue with rheumatologists, and appreciated when their personal experiences of the disease were considered during discussions about symptom relief and treatment options. The study also mentions the psychological challenges faced by individuals with RA, such as distress and helplessness, and the need for acceptance and support from healthcare providers. Collaborative teamwork and clear communication were identified as factors that contributed to patients' overall well-being and disease management. The study recommends that healthcare providers ensure that patients with RA have a comprehensive understanding of their treatment plan, expectations, and available support through effective communication and education.

Psychological well-being has been found to have a negative correlation with joint tenderness in individuals with RA (8). In meetings with rheumatologists, a holistic approach is needed to be able to address issues related to one's well-being. The diagnosis of RA often elicits feelings that are overwhelming, a sense that life has taken an unexpected and unwanted turn, and sadness, anxiety, and fear for the future, necessitating professional support (23). While the immediate focus typically revolves around finding the right drug therapy for inflammation and pain reduction, psychological needs should also be addressed. Some participants in this study expressed frustration at not being seen as individuals, but rather as bodies to be examined, a practice that goes against the recommendations (22).

Expanding on the importance of self-management, addressing the various needs of individuals with RA – such as information, emotions, social support, and practical guidance – is crucial (24, 25). Prior research and guidelines have emphasized the value of education and counseling to empower patients and enhance emotional well-being while providing guidance on managing symptoms like pain, fatigue, depression, anxiety, and sleep issues (9-12, 23, 26). Our study highlights that individuals with a disease might feel alone and disappointed due to insufficient attention to their overall well-being. To address this, healthcare professionals should clarify their roles and discuss expectations with patients to ensure comprehensive support. Additional healthcare contacts focusing on well-being may benefit these patients. An unclear understanding of the physician's role contributes to dissatisfaction, emphasizing the need to clarify different healthcare professionals' roles. While participants did not complain about the medical treatment, some experienced negative patient-physician interactions. Minor changes, such as including interested responses about the patient's experience, could improve the quality of these interactions.

Determining the right RA treatment is challenging due to the chronic nature of the disease. Self-management with digital tools offers a promising approach to address RA patients' needs, enhance health outcomes, and boost satisfaction with healthcare interactions (27). Our participants expressed that their well-being varies day-to-day due to symptoms and treatment effects. They bear the responsibility of managing their daily lives, including handling pain, fatigue, and medications. Thus, much of the health-promoting activities for RA patients take place outside the rheumatologist's office. Participants valued healthcare personnel

For peer review only - http://bmjopen.bmj.com/site/about/guidelines.xhtml

considering their observations in disease follow-up. However, some had disappointing experiences. Therefore, we would like to argue that strengthening self-management by using a digital tool to keep a daily log on what happens between visits should be promoted by healthcare, not by the person with RA. From the healthcare perspective, digital tools for self-management may increase the efficiency and time spent during visits, improving relationships with patients, and using more precise data to guide treatment decisions (6).

Various digital tools can enhance access to care in regions lacking specialized teams for this patient group. They can expedite urgent care, boost effectiveness through closer follow-up via web-based education, videoconferencing, telephone, or electronic messaging, as successfully seen in the oncology services (28). It has also increased in healthcare services, in general, during the COVID-19 pandemic (29). A systematic review assessed whether virtual-care options can replace in-person rheumatologist visits while maintaining comparable care quality for RA patients. They found limited evidence on the impact of virtual rheumatology care, with no significant differences in patient outcomes between care provided by rheumatologists and rheumatology nurses. Virtual care offered additional benefits, including improved treatment adherence, functional status, and quality of life (30). However, it is critical that those digital tools are evidence-based and focus on self-management interventions that are developed by healthcare providers and persons with RA to support and empower individuals (31).

Strengths and limitations

This study aimed to understand how patients view healthcare interactions and the impact of a self-care application on patient-doctor communication. However, the findings may only be applicable to this specific disease group at their stage of the disease. The study excluded participants who did not use the app, which may limit the generalizability of the results.

There are potential limitations in self-reporting bias and subjectivity in an interview-based study. To address these limitations, the researchers encouraged honest and candid responses, maintained confidentiality, reflected on their own perspectives and biases, sought input from colleagues, and used analytical frameworks to improve objectivity in data analysis.

For peer review only

CONCLUSION

The study emphasizes the importance of healthcare providers, including physicians and nurses, addressing areas of patient dissatisfaction. It is important for them to actively listen to the patient's concerns, enhance communication, and adopt a patient-centered approach. By addressing these issues, healthcare providers can improve the overall patient experience and satisfaction.

As technology becomes a permanent fixture in our society and healthcare, we must consider how to effectively integrate digital tools. This involves striking a balance between digital and physical meetings, patient preparation, and the healthcare service's role in treatment. The gap between patient expectations and healthcare implementation may explain their feelings of being unheard. The next phase should focus on implementing digital tools in healthcare services to establish a shared understanding between patients and healthcare providers. In this particular setting, it is essential to emphasize the pivotal role of collaborative engagement between patients and healthcare providers.

For peer review only - http://bmjopen.bmj.com/site/about/guidelines.xhtml

Funding

This project was supported by Vinnova, Innovationsfonden and The Research Council of Norway, under the frame of Nordforsk (Grant agreement no. 90825, Project NORA). The funding agreement ensured the authors' independence in designing the study, interpreting the data, and writing and publishing the report.

Competing interests

Karin Schölin Bywall and Jennifer Viberg Johansson have no conflicts of interest to declare. Hanna Blyckert is employed at Elsa Science as a Health educator and User researcher.

Data availability statement

No additional data available.

Ethics approval and consent to participate

The study was approved by the Swedish Ethical Review Authority (Dnr: 2021-05431-01). Consent to participate, both oral and written, was obtained from all participants. This work was carried out in accordance with the Declaration of Helsinki and prioritized participants' privacy and safety. When requesting consent, the participants were informed that they could withdraw from the study at any time with no explanation and that withdrawal would not affect their current treatment. All participants' names were replaced with codes; all personal identifiers were removed so that the persons described, or narratives, were not identifiable.

Contributors

JVJ, HS and KSB conceptualized the study. HB performed the interviews. JVJ and HB analyzed the interviews and interpreted the data. JVJ was the major contributor in writing the manuscript. HS and KSB revised the manuscript substantively. All the authors read and approved the final manuscript.

Acknowledgements

Our greatest gratitude goes out to all the participants who participated in the interviews. Thank you for your time and for sharing your experiences and opinions so generously.

For peer review only - http://bmjopen.bmj.com/site/about/guidelines.xhtml

References

1. Conaghan PG, Green MJ, Emery P. Established rheumatoid arthritis. Best Pract Res Clin Rheumatol. 1999;13(4):561-75.
2. Smolen JS, Aletaha D, McInnes IB. Rheumatoid arthritis. Lancet. 2016;388(10055):2023-38.
3. Alamanos Y, Voulgari PV, Drosos AA, editors. Incidence and prevalence of rheumatoid arthritis, based on the 1987 American College of Rheumatology criteria: a systematic review. Seminars in arthritis and rheumatism; 2006: Elsevier.
4. Aletaha D, Neogi T, Silman AJ, Funovits J, Felson DT, Bingham CO, 3rd, et al. 2010 Rheumatoid arthritis classification criteria: an American College of Rheumatology/European League Against Rheumatism collaborative initiative. Arthritis Rheum. 2010;62(9):2569-81.
5. Majithia V, Geraci SA. Rheumatoid Arthritis: Diagnosis and Management. Am J Med. 2007;120(11):936-9.
6. Mollard E, Michaud K. Self-Management of Rheumatoid Arthritis: Mobile Applications. Current Rheumatol Rep. 2020;23(1):2.
7. Treharne GJ, Lyons AC, Booth DA, Mason SR, Kitas GD. Reactions to disability in patients with early versus established rheumatoid arthritis. Scand J Rheumatol. 2004;33(1):30-8.
8. Gettings L. Psychological well-being in rheumatoid arthritis: a review of the literature. Musculoskelet Care. 2010;8(2):99-106.
9. Smolen JS, Landewé RBM, Bijlsma JWJ, Burmester GR, Dougados M, Kerschbaumer A, et al. EULAR recommendations for the management of rheumatoid arthritis with synthetic and biological disease-modifying antirheumatic drugs: 2019 update. J Ann Rheum Dis. 2020;79(6):685-99.
10. Larsson I, Bremander A, Andersson M. Patient Empowerment and Associations with Disease Activity and Pain-Related and Lifestyle Factors in Patients With Rheumatoid Arthritis. ACR Open Rheumatol. 2021;3(12):842-9.
11. Donnelly S, Manning M, Mannan H, Wilson AG, Kroll T. Renegotiating dimensions of the self: A systematic review and qualitative evidence synthesis of the lived experience of self-managing rheumatoid arthritis. Health Expect. 2020;23(6):1388-411.
12. Zuidema RM, Repping-Wuts H, Evers AWM, Van Gaal BGI, Van Achterberg T. What do we know about rheumatoid arthritis patients' support needs for self-management? A scoping review. Int J Nurs Stud. 2015;52(10):1617-24.
13. Perry B, Geoghegan C, Lin L, McGuire FH, Nido V, Grabert B, et al. Patient preferences for using mobile technologies in clinical trials. Contemp Clin Trials Commun. 2019;15:100399.
14. Nikiphorou E, Santos EJF, Marques A, Böhm P, Bijlsma JW, Daien CI, et al. 2021 EULAR recommendations for the implementation of self-management strategies in patients with inflammatory arthritis. J Ann Rheum Dis. 2021;80(10):1278-85.
15. Bodenheimer T, Lorig K, Holman H, Grumbach K. Patient Self-management of Chronic Disease in Primary Care. JAMA. 2002;288(19):2469-75.
16. Von Korff M, Tiemens B. Individualized stepped care of chronic illness. West J Med. 2000;172(2):133-7.
17. Kvale S, Brinkmann S. Interviews: Learning the craft of qualitative research interviewing: Sage; 2009.
18. Krueger RA, Casey MA. Focus groups: A practical guide for applied research: Sage Publications; 2014.
19. ATLAS.ti Scientific Software Development GmbH. ATLAS.ti Web - The easiest way to uncover qualitative insights 2022 [Available from: https://atlasti.com/atlas-ti-web.
20. Burnard P. Writing a qualitative research report. Nurs Educ Today. 2004;24(3):174-9.

For peer review only - http://bmjopen.bmj.com/site/about/guidelines.xhtml

21. Burnard P, Gill P, Stewart K, Treasure E, Chadwick B. Analysing and presenting qualitative data. Brit Dent J. 2008;204(8):429-32.
22. Nagy G, Roodenrijs NMT, Welsing PMJ, Kedves M, Hamar A, van der Goes MC, et al. EULAR points to consider for the management of difficult-to-treat rheumatoid arthritis. Ann Rheum Dis. 2022;81(1):20-33.
23. Hehir M, Carr M, Davis B, Radford S, Robertson L, Tipler S, et al. Nursing support at the onset of rheumatoid arthritis: Time and space for emotions, practicalities and self-management. Musculoskelet Care. 2008;6(2):124-34.
24. Radford S, Carr M, Hehir M, Davis B, Robertson L, Cockshott Z, et al. 'It's quite hard to grasp the enormity of it': Perceived needs of people upon diagnosis of rheumatoid arthritis. Musculoskelet Care. 2008;6(3):155-67.
25. Jones B, Bennett S, Larsson I, Zangi H, Boström C, Van der Elst K, et al. Disseminating and assessing implementation of the EULAR recommendations for patient education in inflammatory arthritis: a mixed-methods study with patients' perspectives. J RMD Open. 2022;8(1):e002256.
26. Knittle K, Maes S, de Gucht V. Psychological interventions for rheumatoid arthritis: Examining the role of self-regulation with a systematic review and meta-analysis of randomized controlled trials. Arthritis Care Res. 2010;62(10):1460-72.
27. Najm A, Lempp H, Gossec L, Berenbaum F, Nikiphorou E. Needs, Experiences, and Views of People With Rheumatic and Musculoskeletal Diseases on Self-Management Mobile Health Apps: Mixed Methods Study. JMIR Mhealth Uhealth. 2020;8(4):e14351.
28. Sirintrapun SJ, Lopez AM. Telemedicine in Cancer Care. 2018(38):540-5.
29. Wosik J, Fudim M, Cameron B, Gellad ZF, Cho A, Phinney D, et al. Telehealth transformation: COVID-19 and the rise of virtual care. J Am Med Informat Assoc. 2020;27(6):957-62.
30. Han L, Hazlewood GS, Barnabe C, Barber CEH. Systematic Review of Outcomes and Patient Experience With Virtual Care in Rheumatoid Arthritis. Arthritis Care Res. 2022;74(9):1484-92.
31. Rodríguez Sánchez-Laulhé P, Luque-Romero LG, Barrero-García FJ, Biscarri-Carbonero Á, Blanquero J, Suero-Pineda A, et al. An Exercise and Educational and Self-management Program Delivered With a Smartphone App (CareHand) in Adults With Rheumatoid Arthritis of the Hands: Randomized Controlled Trial. JMIR Mhealth Uhealth. 2022;10(4):e35462.

For peer review only

Example of App Content: Explore snapshots of the app's features. For more details, please visit: https://www.elsa.science/en/

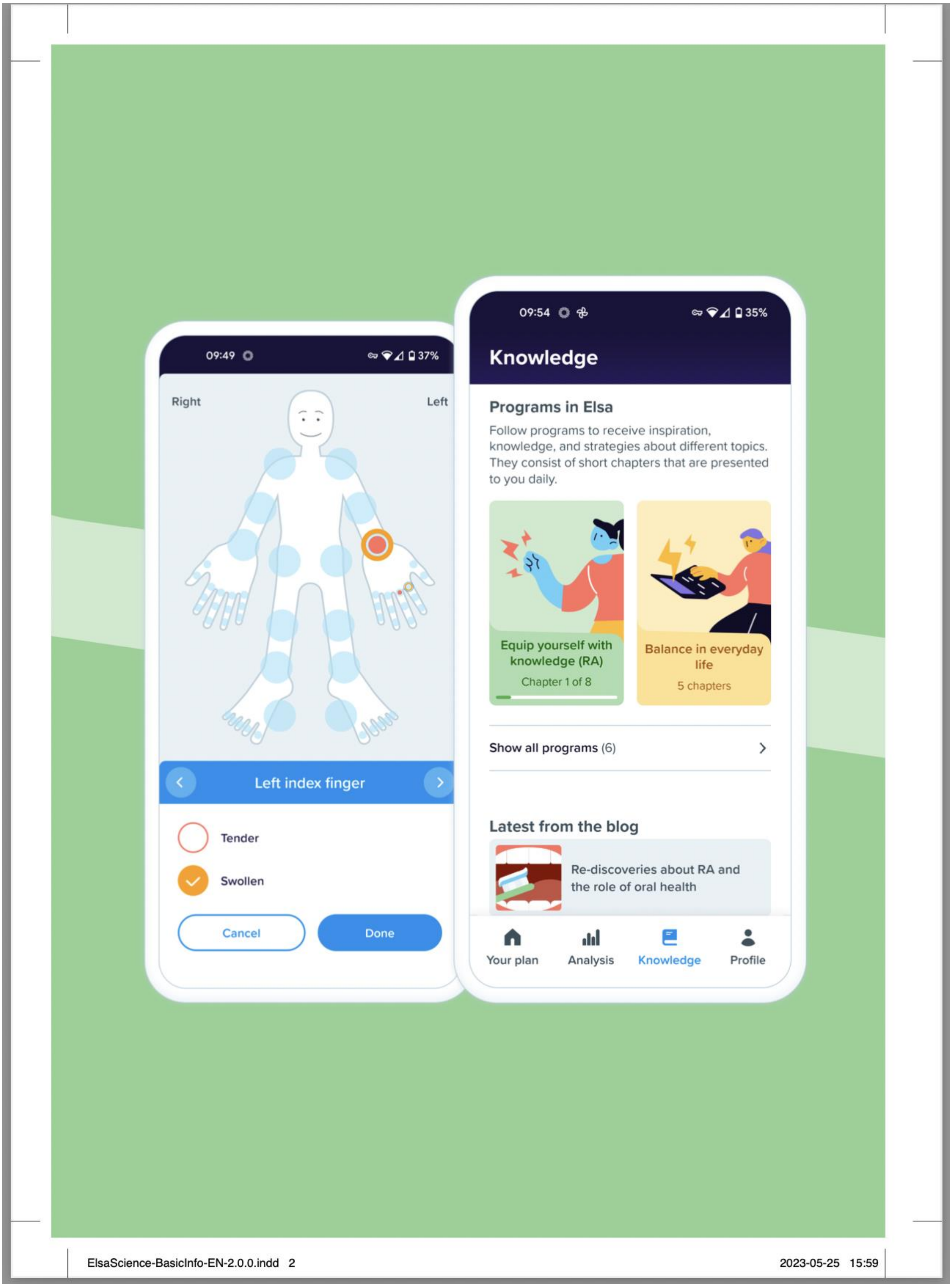

ELSA SCIENCE

The Self-Care App

Rheumatic disease? This is for you.

The Elsa Science Self-Care App is your partner in managing rheumatic conditions at any stage of the disease so you can focus on quality of life.

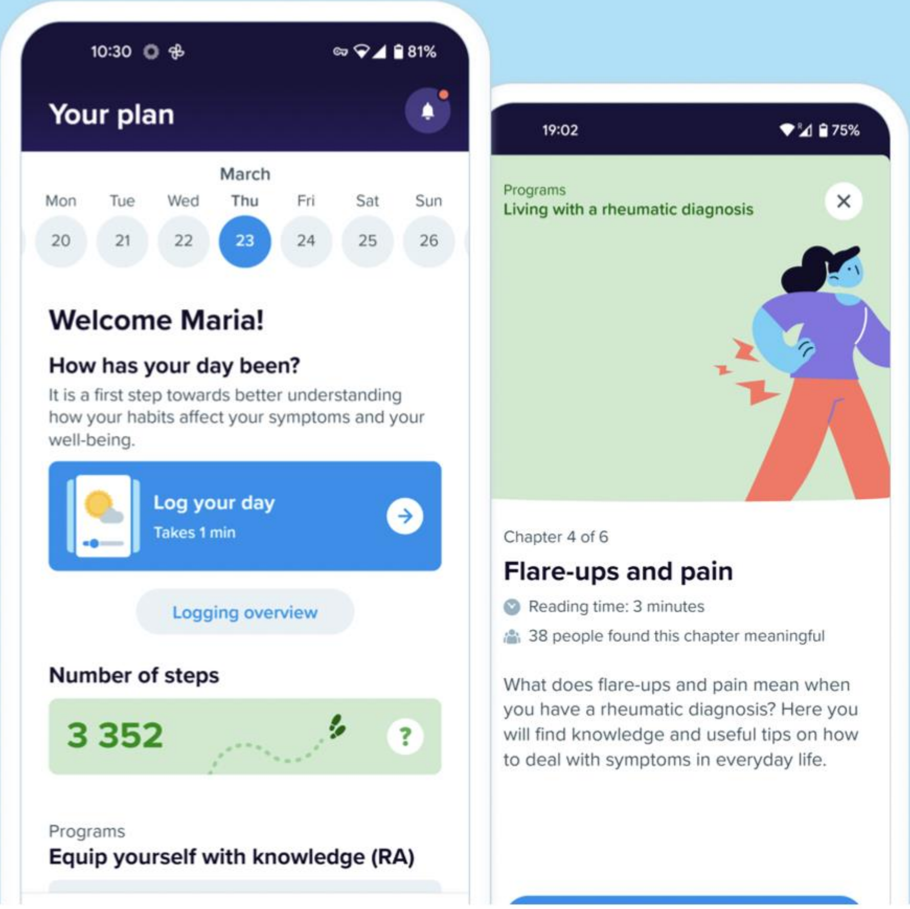

ElsaScience-BasicInfo-EN-2.0.0.indd 1 2023-05-25 15:59

COREQ (COnsolidated criteria for REporting Qualitative research) Checklist

A checklist of items that should be included in reports of qualitative research. You must report the page number in your manuscript where you consider each of the items listed in this checklist. If you have not included this information, either revise your manuscript accordingly before submitting or note N/A.

Topic	Item No.	Guide Questions/Description	Reported on Page No.
**Domain 1: Research team and reflexivity**			
Personal characteristics			
Interviewer/facilitator	1	Which author/s conducted the interview or focus group?	
Credentials	2	What were the researcher's credentials? E.g. PhD, MD	
Occupation	3	What was their occupation at the time of the study?	
Gender	4	Was the researcher male or female?	
Experience and training	5	What experience or training did the researcher have?	
Relationship with participants			
Relationship established	6	Was a relationship established prior to study commencement?	
Participant knowledge of the interviewer	7	What did the participants know about the researcher? e.g. personal goals, reasons for doing the research	
Interviewer characteristics	8	What characteristics were reported about the inter viewer/facilitator? e.g. Bias, assumptions, reasons and interests in the research topic	
**Domain 2: Study design**			
Theoretical framework			
Methodological orientation and Theory	9	What methodological orientation was stated to underpin the study? e.g. grounded theory, discourse analysis, ethnography, phenomenology, content analysis	
Participant selection			
Sampling	10	How were participants selected? e.g. purposive, convenience, consecutive, snowball	
Method of approach	11	How were participants approached? e.g. face-to-face, telephone, mail, email	
Sample size	12	How many participants were in the study?	
Non-participation	13	How many people refused to participate or dropped out? Reasons?	
Setting			
Setting of data collection	14	Where was the data collected? e.g. home, clinic, workplace	
Presence of non-participants	15	Was anyone else present besides the participants and researchers?	
Description of sample	16	What are the important characteristics of the sample? e.g. demographic data, date	
Data collection			
Interview guide	17	Were questions, prompts, guides provided by the authors? Was it pilot tested?	
Repeat interviews	18	Were repeat inter views carried out? If yes, how many?	
Audio/visual recording	19	Did the research use audio or visual recording to collect the data?	
Field notes	20	Were field notes made during and/or after the inter view or focus group?	
Duration	21	What was the duration of the inter views or focus group?	
Data saturation	22	Was data saturation discussed?	
Transcripts returned	23	Were transcripts returned to participants for comment and/or	

For peer review only

Topic	Item No.	Guide Questions/Description	Reported on Page No.
		correction?	
**Domain 3: analysis and findings**			
Data analysis			
Number of data coders	24	How many data coders coded the data?	
Description of the coding tree	25	Did authors provide a description of the coding tree?	
Derivation of themes	26	Were themes identified in advance or derived from the data?	
Software	27	What software, if applicable, was used to manage the data?	
Participant checking	28	Did participants provide feedback on the findings?	
Reporting			
Quotations presented	29	Were participant quotations presented to illustrate the themes/findings? Was each quotation identified? e.g. participant number	
Data and findings consistent	30	Was there consistency between the data presented and the findings?	
Clarity of major themes	31	Were major themes clearly presented in the findings?	
Clarity of minor themes	32	Is there a description of diverse cases or discussion of minor themes?	

Developed from: Tong A, Sainsbury P, Craig J. Consolidated criteria for reporting qualitative research (COREQ): a 32-item checklist for interviews and focus groups. *International Journal for Quality in Health Care*. 2007. Volume 19, Number 6: pp. 349 – 357

Once you have completed this checklist, please save a copy and upload it as part of your submission. DO NOT include this checklist as part of the main manuscript document. It must be uploaded as a separate file.

For peer review only
